# The *Caenorhabditis elegans* p38 MAPK Gene plays a key role in protection from mycobacteria

**DOI:** 10.1002/mbo3.341

**Published:** 2016-02-25

**Authors:** Thushara Galbadage, Tonya F. Shepherd, Suat L. G. Cirillo, Tina L. Gumienny, Jeffrey D. Cirillo

**Affiliations:** ^1^ Departments of Microbial Pathogenesis and ImmunologyTexas A&M Health Science CenterBryanTexas77807‐3260; ^2^ Department of BiologyTexas Woman's UniversityDentonTexas76204‐5799

**Keywords:** *C. elegans*, innate immunity, *Mycobacterium*

## Abstract

Mitogen‐activated protein kinases (MAPK) are critical mediators of cellular responses to pathogens and are activated in response to infection, but investigation is difficult in multi‐cell hosts due to developmental lethality of mutations. *Mycobacterium marinum* (*Mm*) is an established model for tuberculosis, a disease afflicting nearly one‐third of the world's population. We found that *Mm*‐infected *Caenorhabditis elegans* display >80% mortality, but nonpathogenic *M. smegmatis* cause <15% mortality. *C. elegans* display pathological changes when infected with *Mm,* whereas *Mm* mutants produce lower mortality, suggesting that *C. elegans* is a promising virulence model for detailed genetic analysis. *C. elegans* MAPK mutants are hypersusceptible to mycobacterial infection; however, the *C. elegans* TOL‐like, TGF‐*β* and insulin‐like pathway genes do not play important roles in susceptibility. We show that pathogenic mycobacteria inhibit MAPK‐mediated protection through the MAPK phosphatase gene and demonstrate that *C. elegans* provide a genetically tractable pathogenicity model of both the host and pathogen.

## Introduction

The innate immune response acts as a first line of defense in protecting the host against pathogens and activates the adaptive response by producing specific cytokines and chemokines (Iwasaki and Medzhitov 2010). Pattern recognition receptors (PRRs) recognize conserved structures not present in eukaryotic cells and provide partial protection until the adaptive immune system takes over (Janeway 1992). Several classes of PRRs can trigger innate immunity, including C‐type lectin receptors (CLRs), tol‐like receptors (TLRs), and NOD‐like receptors (NLRs) (Kleinnijenhuis et al. 2011; Kingeter and Lin 2012). All identified PRRs activate the p38 mitogen‐activated protein kinase (MAPK) pathway in response to pathogen‐associated molecular patterns (PAMPs) (Kawai and Akira 2010), suggesting that p38 MAPK is a key mediator of immunity (Koul et al. 2004). Mycobacteria are some of the most important pathogens worldwide, encompassing the causative agents of leprosy and tuberculosis. Tuberculosis (TB) causes ~1.4 million deaths annually, latently infecting a third of the world's population and multidrug‐resistant (MDR‐TB) strains are now prevalent in all continents (Glaziou et al. 2013). While many interventions focus on modulating the adaptive immune system, the innate immune response is also critical for host protection (Pan et al. 2005).

The innate immune response is initially activated by macrophages at the site of mycobacterial infection (Roach and Schorey 2002; Kleinnijenhuis et al. 2011). The T‐cell‐mediated adaptive response is activated shortly thereafter and can reduce the bacterial load (Stenger and Modlin 1999). Pathogenic mycobacteria activate all three major MAPK pathways, but there is little direct evidence for a role in pathogenesis due to the detrimental effects of mutations during development in mice. When mycobacteria infect macrophages, ERK and p38 MAPK are activated by phosphorylation, inducing an inflammatory response (Schorey and Cooper 2003). Mycobacterial lipoarabinomannan (LAM) acts as a PAMP that activates the innate immune response through MAPK (Strohmeier and Fenton 1999). LAM on the surface of pathogenic mycobacteria is mannose‐capped (Man‐LAM); whereas, nonpathogenic mycobacteria have arabinosylated LAM (Ara‐LAM). Ara‐LAM induces cytokines and inflammatory mediators, whereas Man‐LAM promotes dephosphorylation that suppresses MAPK activation and, thereby, inflammatory cytokines (Roach et al. 1993; Knutson et al. 1998; Juffermans et al. 2000). Despite the differences in activation of MAPK and the inflammatory response, the mechanisms for suppression of MAPK and their significance during disease remain elusive.

Genetically tractable virulence models are valuable for pathogenesis studies, since they allow rapid analysis of complex interactions often difficult to study in mammals. Several virulence models have been used for study of mycobacteria, including tissue culture cells, mice, guinea pigs, and rabbits (Bishai et al. 1999; Jain et al. 2007), but can be costly and difficult to genetically manipulate. *C. elegans* is a valuable model for study of host responses because it has a well‐characterized genome and an extensive array of molecular tools (Ashrafi et al. 2003; Irazoqui et al. 2010). The innate immune response and host–pathogen interactions can be analyzed in great detail using *C. elegans* (Garsin et al. 2001; Couillault and Ewbank 2002; Irazoqui et al. 2010). *C. elegans* use reactive oxygen species (ROS) and lysosomes to defend against invading pathogens, similar to mammalian macrophages and neutrophils (Mallo et al. 2002; Chavez et al. 2007). The *C. elegans* p38 isoforms of MAPKs, designated *pmk‐1‐3*, are localized to the intestine and are important in the response to pathogenic bacteria (Coleman and Mylonakis 2009; Shivers et al. 2009). Many such responses are mediated by PMK‐1, the best characterized of the p38 isoforms (Aballay et al. 2003; Kim et al. 2004; Jebamercy et al. 2013), making it potentially important for a host response to pathogenic mycobacteria. *Mycobacterium marinum* (*Mm)* is commonly used as a model for *M. tuberculosis* due to its rapid growth rate and ease of use (Shiloh and Champion 2010).

We combined the model organisms *Mm* and *C. elegans* to analyze the role of MAPK in mycobacterial pathogenesis. We found that infection of *C. elegans* with *Mm* causes higher morbidity and mortality than the nonpathogen *M. smegmatis* (Ms). *Mm*‐infected *C. elegans* undergo irreversible pathological changes that lead to nematode death. Mortality of *C. elegans* correlates well with virulence of several *Mm* mutants, suggesting that the nematode serves as a useful virulence model. We found that *C. elegans* protection is controlled by the p38 MAPK pathway and *Mm* can hijack MAPK phosphatase to interfere with protection. These studies demonstrate that *C. elegans* has particular value for study of MAPK, which can be difficult to study in mammals and has provided insight into the mechanisms that lead to mycobacterial pathogenesis.

## Materials and Methods

### Bacterial strains and growth conditions

A wild‐type clinical isolate of *Mm* strain M, wild‐type *Ms* (mc^2^155), and *E. coli* (OP50) were used. Two constitutively expressing tdTomato fluorescent mycobacterial strains (ψmm91 and ψms23) were derived by transforming *Mm* and *Ms*, respectively, with pJDC60 (pFJS8ΔGFP::tdTomato expressed by L5 promoter, Kan^R^). *M. marinum* cultures were grown at 32°C standing in T25 tissue culture flasks. *E. coli* and *M. smegmatis* cultures were grown at 37°C shaking in sterile disposable glass test tubes. *M. marinum* and *M. smegmatis* were grown in Middlebrook 7H9 media (Difco, Sparks, MD) supplemented with 0.5% glycerol, 10% albumin‐dextrose complex (ADC), and 0.25% Tween 80 (M‐ADC‐TW), whereas *E. coli* was grown in Miller's Luria Broth (NPI, Mt. Prospect, IL).

### *C. elegans* strains

The *C. elegans* strains were obtained from the *Caenorhabditis* Genetics Center (CGC), University of Minnesota. The wild‐type strain was N2 and mutants included: TP12 [*kaIs12*(col‐19::GFP)], GR1307 [*daf‐16*(*mgDf50*)], NU3 [*dbl‐1*(nk3)], KU25 [*pmk‐1*(km25)], EU31 [*skn‐1*(zu135)], IG10 [*tol‐1*(nr2033)], and JT366 [*vhp‐1*(sa366)]. Nematodes were grown and maintained on nematode growth media (NGM) plates using standard methods at 19°–21°C (Brenner 1974). Synchronous cohorts of *C. elegans* were obtained by lysing gravid nematodes using an alkaline bleach solution (Emmons et al. 1979). After removing the bleach solution and washing the embryos, they were stored in M9 buffer overnight to obtain L1 larvae. These L1 larvae were transferred on NGM plates seeded with OP50 *E. coli* for growth of age‐synchronized nematodes.

### *C. elegans* infection

*E. coli*,* Ms* and *Mm* grown to stationary phase were seeded on tissue culture dishes with NGM agar. Three‐day‐old age‐synchronized adult *C. elegans* were infected for 4, 24, or 48 h with each bacterial strain. Volumes of 70 *μ*L for *E. coli*,* M. smegmatis,* or *M. marinum* were seeded on small tissue culture dishes (35 × 10 mm, Falcon), with NGM agar by spreading the bacterial cultures to cover over 3/4th of the infection plates. Infection plates were placed at room temperature overnight to allow bacterial cultures to grow, equilibrate/stabilize and be ready for infection the next day. Three‐day‐old age‐synchronized adult *C. elegans* were washed with ddH_2_O to remove residual *E. coli* on their surface and transferred onto plates seeded with *E. coli, M. smegmatis, or M. marinum* for infection. Cohorts of *C. elegans* were exposed for a period of 4, 24, or 48 h to each individual bacterial strain. After infection, nematodes were transferred onto NGM plates seeded with *E. coli* and assessed for pathology and mortality. After the period of infection, the nematodes were transferred onto small NGM recovery plates with *E. coli* seeded in the center of each plate. A total of 20 nematodes were incubated per small recovery NGM plate and cohorts of 60 nematodes were used for each bacterial infection. Nematodes were counted daily and transferred onto fresh *E. coli* seeded plates every other day until experimental nematodes stopped laying eggs. Then they were transferred every 3–4 days to avoid overgrowth of seeded *E. coli* until the nematodes reached senescence and died. The nematodes were considered dead if they were unresponsive to touch with a wire pick. Bacterial infection experiments were repeated at least three times with 20, 40, or 60 nematodes, unless stated otherwise.

### Survival and morphological characterization of *C. elegans*

*C. elegans* L1 larvae incubated on *E. coli* seeded NGM plates after synchronization were considered 0 days old. They were grown for 3 days at room temperature before subjection to bacterial infection. On day 4, they were recovered on to fresh *E. coli* seeded NGM plates and followed up for survival and changes in morphology. Number of nematodes that died due to bagging of the adult nematode (where embryos hatch within the adult and cause the death of the adult) on day 5 and 6 were counted. Nematodes that lost their dark pigmentation after bacterial infection were characterized as depigmented. Nematodes that were less than 2/3rd the length of a healthy nematode were characterized as having a shortened length. On day 6, depigmented and shorter nematodes were counted. Nematodes that died prior to 15 days were considered to have a shortened lifespan.

### Imaging of *C. elegans* for morphological differences and fluorescent mycobacteria

TP12 [*kaIs12*(*col‐19*::GFP)] *C. elegans* expressing cuticular eGFP‐tagged *col‐19*, a member of the collagen superfamily, were infected with ψms23 (*Ms*::tdTomato) or ψmm91 (*Mm*::tdTomato). Exposed *C. elegans* were mounted on a 3% agar pad with a thickness of two labeling tapes. 15 *μ*L of 5 mmol/L levamisole was added to immobilize the nematodes and keep the agar pad moist for imaging. 25‐mm round cover slips with a thickness of 0.17 mm (#1.5) were placed on the agar pad to keep *C. elegans* in place and for imaging. Using a confocal microscope (Nikon A1R+), exposed nematodes were imaged under differential interface contrast (DIC) and fluorescent filters (FITC and TRITC) using a 10× objective. The nematodes were imaged at 4, 12, 18, and 24 h during infection, and 6 and 24 h post infection. The 60 nematodes for each bacterial infection and time point were imaged. DIC images were used to identify patterns of morphological change in cohorts of nematodes infected with different bacteria. Morphological changes in *M. smegmatis* and *M. marinum* exposed nematodes were compared with that of *E. coli* exposed nematodes. Fluorescent imaging using FITC and TRITC filters was used to determine localization during colonization of *M. marinum* and *M. smegmatis* within *C. elegans*. The 60 nematodes imaged for each time point were randomly divided into three groups and mycobacterial fluorescent signal determined. Three segments of each nematode were imaged (upper, middle, and lower) and fluorescent bacteria were quantified within each defined area imaged.

### CFU assay for bacterial load and colonization of *C. elegans*

Nematodes were transferred and colony forming units (CFU) determined by homogenization and plating dilutions. At 4, 12, and 24 h during infection and 6 h post infection, a total of 30 *C. elegans* infected with wild‐type *M. marinum* or *M. smegmatis* were transferred to 500 *μ*L of M9 buffer with 0.05% Tween‐20 in 1.5 mL microcentrifuge tubes. After briefly vortexing, they were spun down at 800 rpm for 15 sec and the supernatant removed. These nematodes were washed three times with 500 *μ*L of 1× PBS with 0.05% Tween‐20 and resuspend in 600 *μ*L of 1× PBS with 0.05% Tween‐20 and 100 *μ*L plated for levels of bacteria in serial dilutions to determine background. The nematodes remaining in 500 *μ*L of 1× PBS with 0.05% Tween‐20 were homogenized with a hand held motorized pestle for a total of 45 sec. After the nematodes were broken up, releasing the bacteria, they were vortexed on high and plated for CFU in serial dilutions. A quantity of 20 *μ*L spot plating was carried out in triplicate for each of the dilutions. *M. smegmatis* was grown at 37°C and *M. marinum* was grown at 32°C overnight.

### Macrophage infection mutant (MIM) *M. marinum* infection

Nematodes were infected with *M. marinum* mutants that were constructed previously and are known to impact infection of mammalian macrophages. Similar methods were used to those described for wild‐type *M. marinum*. *Mm* MIMs, *mimA‐K, nrp, ppe24, sdhD, pks12, fadD29, fadD30*, and *ppe53,* were constructed by insertion of kanamycin resistance within the coding region of each gene (Mehta et al. 2006). Three genes were complemented with their respective *Mtb* gene orthologs (*mimA*::Rv0246, *mimG*::Rv3242c, *mimI*::Rv1502), as described previously (Mehta et al. 2006). We used these mutants and exposed 3 day old adult N2 nematodes for a period of 24 h. MIM cultures were grown until stationary phase of growth (OD >1.2) and 70 *μ*L of each were seeded on small NGM plates, the day prior to infection. Triplicates of 20 N2 worms (total of 60) were infected with each strain of *M. marinum*. They were then allowed to recover and mortality rates assessed 2 days post‐infection (day 6). To confirm the attenuation was due to a specific gene in *M. marinum*, three strains complemented with their *M. tuberculosis* orthologs were also used to expose *C. elegans* and mortality rates were compared, 2 days post infection (day 6).

### RNAi knock‐down and confirmation

After bleaching was used to age‐synchronize *C. elegans*, N2 L1 larvae were inoculated on small NGM plates seeded with *E. coli* strain HT115 (DE3) with RNAi constructs cloned in the pL4440‐DEST vector and selected with ampicillin resistance (100 *μ*g/mL). *E. coli* strains producing dsRNA corresponding to the *C. elegans pmk‐1, tol‐1*,* dbl‐1*,* daf‐16, skn‐1*, and *vhp‐1* genes were used (Table S2). A quantity of 50 *μ*L of *E. coli* carrying the RNAi plasmid was added to the plates on day one for 24 h. Adult N2 knock‐down animals were then infected with wild‐type *M. smegmatis* or *M. marinum* on day three and susceptibility to infection with mycobacteria assessed.

For confirmation, RNA from 3 day old N2 nematodes grown on small NGM plates seeded with *E. coli* strain HT115 (DE3) expressing individual RNAi constructs for *pmk‐1*,* tol‐1*,* dbl‐1*,* daf‐16*,* skn‐1*, and *vhp‐1* was isolated after dissolving the nematodes in Trizol. cDNA was produced using random primers and specific qPCR run for each target gene (Table S3). *Cdc‐42* was used as a housekeeping gene control. Age‐synchronized 3 day old nematodes from each RNAi knock‐down mutant strain used in this study were subjected to RT‐PCR and qPCR confirmation. RNA levels were normalized to housekeeping genes *pmp‐3* and *cdc‐42*, and compared against N2‐infected with *E. coli* expressing the empty RNAi vector.

### High‐resolution confocal imaging of *C. elegans*

Using a confocal microscope (Nikon A1R+) with spectral capability, 25 emission channels with a resolution of 6.0 nm were use to detect the wavelengths of 500–640 nm. This wavelength range was used to detect excitation of GFP (500–520 nm) and tdTomato (560–620 nm). Using a 40× oil immersion objective, TP12 nematodes infected with *E. coli* (OP50), ψms23 (*M. smegmatis*::tdTomato), or ψmm91 (*M. marinum*::tdTomato) were imaged during (4 and 24 h) and post infection (30 h). The nematodes were immobilized with 15 *μ*L of levamisole (5 mmol/L) and the head, mid‐gut region, and lower‐gut regions were imaged. About 15 nematodes for each time point for infection with ψms23 and ψmm91 and about 5 nematodes were imaged for each time point for infection with *E. coli*. Representative images are presented to show differences in bacterial colonization and morphological changes in nematodes after infection.

### Transmission electron microscopy imaging of ***C. elegans***

*C. elegans* infected with bacteria were fixed, embedded, sectioned, and imaged using previously described methods with slight modifications (Hall et al. 2012; Schultz et al. 2014) at 4 and 24 h during infection and 6 h postinfection. Nematodes from *Ms* or *Mm* were embedded and longitudinal sections were obtained using a microtome. *C. elegans* from each group (*E. coli*,* M. smegmatis* and *M. marinum*) were obtained and washed twice with 500 *μ*L of 1 × M9 buffer. The nematodes were then immersed in fixative with 2.5% glutaraldehyde, 2% paraformaldehyde and 0.1% (w/v) malachite green in a working buffer (0.1 HEPES, pH 7.4, containing 2 mmol/L MgCl2). Using a PELCO BioWave^®^ microwave, the nematodes in the fixative solution were microwaved under vacuum at 100 W for 10 min and allowed to stand at room temperature for 3 min. This process was repeated and the specimens were placed at room temperature for 1 h. A second step of microwaving at 500 W for 10 sec, stand for 20 sec and microwaved for 10 sec. The nematodes were washed three times with 500 *μ*L of working buffer and microwaved for 1 min after each wash. To improve contrast, the nematodes were postfixed in 1% (w/v) osmium tetroxide with 1.5% (w/v) potassium ferricyanide in working buffer. They were microwaved at 100 W for 2 min, followed by letting them stand for 2 min. This was repeated four times. The specimens were dehydrated by rinsing the samples with 50% acetone, 70% acetone, 90% acetone, and three times with 100% acetone. After each rinse, the specimens were microwaved at 150 W for 1 min. The specimens were then infiltrated with resin, Quetol 651‐modified Spurr low viscosity epoxy resin, by 1:1 acetone:resin followed by 100% resin three times. They were microwaved at 200 W for 4 min between each infiltration step. The specimens were transferred into embedding tubes and the resin was allowed to polymerize overnight at 60°C. Longitudinal sections were obtained from the embedded specimens using a microtome and poststained with 2% (w/v) aqueous uranyl acetate. A JEOL 1200EX transmission electron microscope at an accelerated voltage of 100 kV was used to image these specimens.

### *C. elegans* mutant confirmation

Mutant confirmation was accomplished using PCR with specific primers. Age‐synchronized N2 L1 larvae were incubated on plates seeded with *E. coli* producing each of the individual dsRNA for the target gene (Table S2). Three‐day‐old adult nematodes were obtained and RNA was extracted after dissolving the nematodes in Trizol. cDNA was produced using random primers and PCR was run for each target gene (Table S3). *Cdc‐42* was used as a housekeeping gene control. Age‐synchronized 3‐day‐old nematodes from each mutant strain used in this study were also subjected to PCR for confirmation.

### Statistical analyses

All experiments were repeated at least twice with consistent results. Parametric two‐tailed unpaired *t*‐test statistical analyzes were used to compare the means of different bacterial infection groups at distinct time points using GraphPad Prism version 5 software (La Jolla, CA, USA). Means and standard deviations or standard errors are presented. Means, standard deviations, and standard errors were calculated using GraphPad Prism 5 software and Microsoft Excel. Nonparametric log‐rank statistics were used to determine the difference in survival for groups of *C. elegans* after infection, using an online application for survival analysis of lifespan assays found on http://sbi.postech.ac.kr/oasis (released on May 2009; last accessed on April 20th 2014) (Yang et al. 2011).

## Results

### Pathogenic mycobacteria cause mortality in *C. elegans*

**Figure 1:**
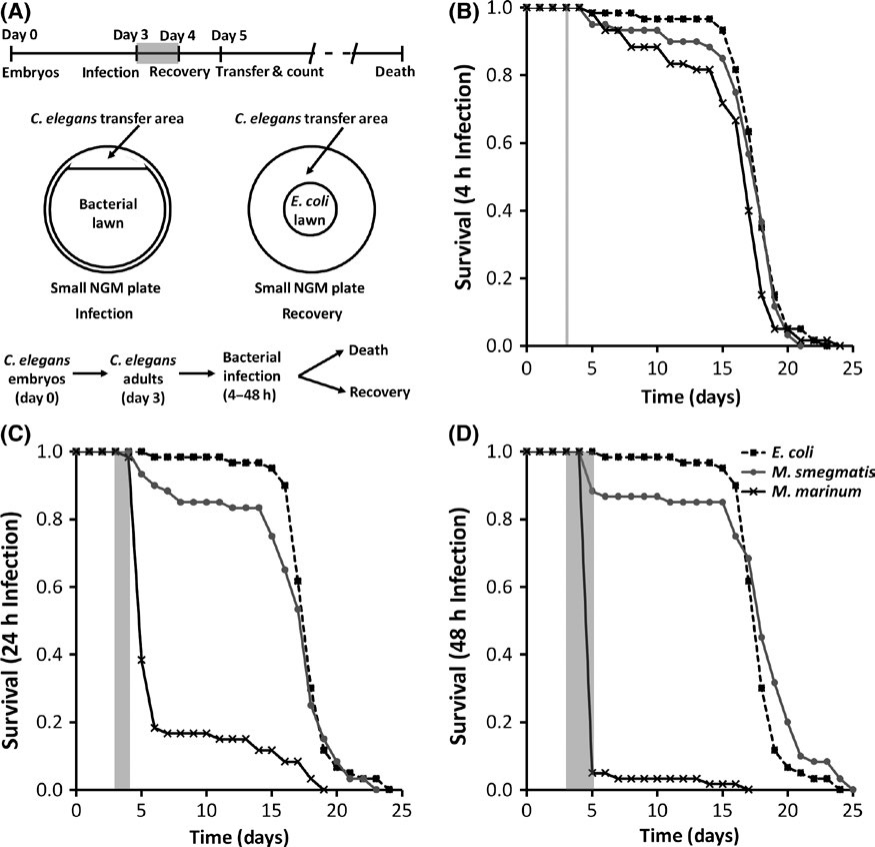
Pathogenic mycobacteria cause *C. elegans* mortality. (A) Procedure for infecting *C. elegans*. Adult nematodes were grown on NGM plates seeded with *E. coli* and infected for (B) 4, (C) 24 or (D) 48 h on day 3 as indicated by the gray shaded regions on each graph. During recovery on NGM plates seeded with *E. coli*, nematode survival was determined. Data shown are survival for 20 nematodes in each group. Key in (D) is for (B–D). Log‐rank analysis was used to compare survival of each infection group. (B) *Mm* versus. *Ms P* = 0.0567; *Mm* versus. *E. coli P* = 0.0095; *Ms* versus *E. coli P* = 0.3776. (C) *Mm* versus *E. coli P* < 0.0001; *Ms* versus *E. coli P* = 0.2207; *Mm* versus *Ms P* < 0.0001. (D) *Mm* versus *Ms P* < 0.0001; *Mm* versus *E. coli P* < 0.0001; *Ms* versus *E. coli P* = 0.0750.

We infected *C. elegans* with *M. smegmatis* (*Ms*), a nonpathogenic mycobacteria, and *Mm*. *C. elegans* use *E. coli* as their natural food, which is harmless for these nematodes and necessary for their survival. We found that *Mm* causes higher rates of mortality in *C. elegans* than *Ms* or *E. coli*. In fact, *Ms* does not display significantly different rates of mortality as compared with *E. coli* where the nematodes die at the normal age of 18–20 days. When infected with *Mm* for 24 h, *C. elegans* display >80% mortality at 2 d post infection; whereas, *Ms* produce <15% mortality (Fig. 1). The majority of nematode deaths in the *Mm*‐infected group occur due to an event described as bagging, where the gravid adult nematode is unable to lay eggs and viviparity (egg hatching) occurs within the worm, resulting in the death of the adult, but survival of progeny. Consistent with these observations, bagging can occur as a *C. elegans* response to bacterial infection (Mosser et al. 2011). Even the <20% of nematodes that survive >24 h after *Mm* infection display pathology (Fig. S1). Longer infections with *Mm* increase the rate and number of mortalities, but do not impact mortality from *Ms*, suggesting that the mechanisms involved are specific to the pathogenic species.

### *Mm* cause irreversible pathological changes in *C. elegans*

**Figure 2:**
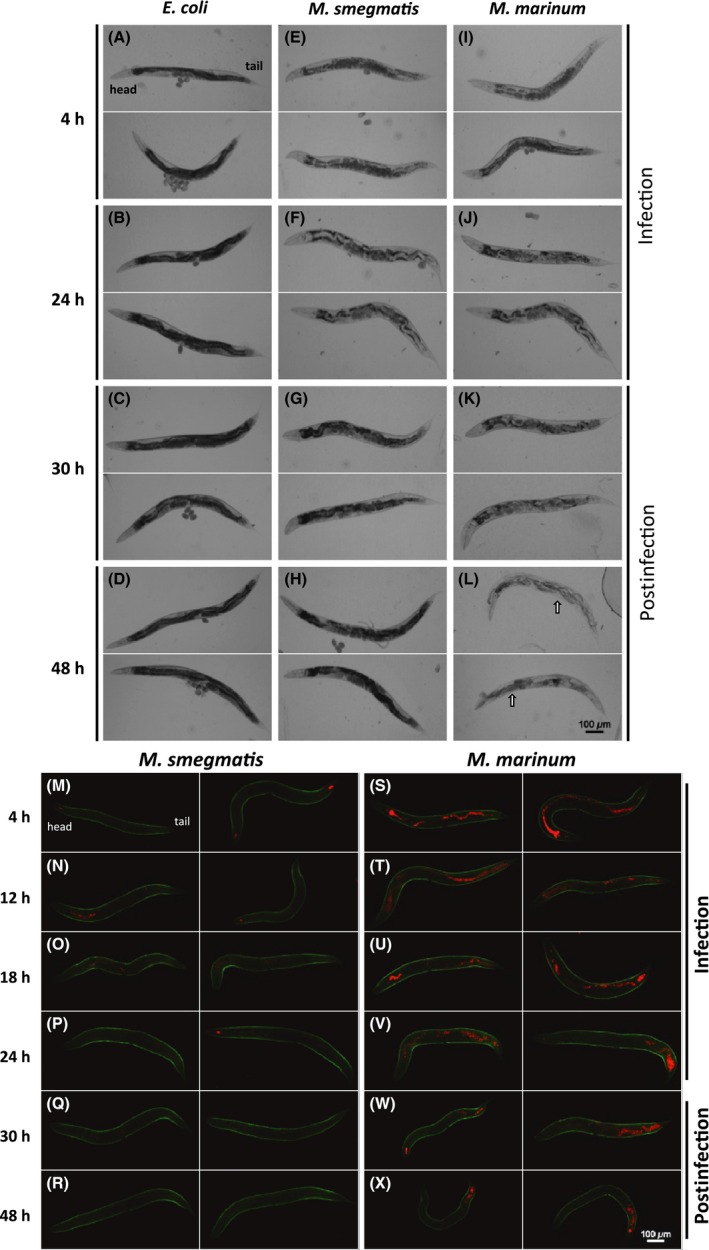
*M. marinum* persists in *C. elegans* and causes irreversible pathology. Morphology of *C. elegans* by light microscopy (A–L) and GFP fluorescent *C. elegans* by confocal microscopy (M–X) infected for 24 h and recovered on *E. coli* 48 h postinfection. (A–D) *E. coli* (OP50) infected nematodes are able to lay eggs and retain their dark pigmentation. (E–H) *M. smegmatis* (tdTomato)‐infected nematodes undergo reversible retention of eggs and loss of pigmentation during infection, but regain pigmentation and the ability to lay eggs postinfection. (I–L) *M. marinum* (tdTomato)‐infected nematodes experience irreversible retention of eggs and loss of pigmentation during infection. (L) The arrows indicate “bagging” morphological changes that occur when embryos are hatched in utero.

*C. elegans* were imaged by light microscopy during infection for 24 h and for 24 h postinfection. Nematodes infected with mycobacteria displayed reduced pigmentation and increased retention of embryos as compared with those infected with *E. coli* (Fig. 2). *E. coli*‐infected nematodes did not lose pigmentation or display pathology and lay eggs (Fig. 2A–D, S2). In the case of *Ms*, nematodes initially lose pigmentation and retain embryos*,* but regain pigmentation and lay eggs within 6 h post infection (Fig. 2E–H). In contrast to *Ms*, nematodes infected with *Mm* undergo irreversible pathological changes and are unable to lay eggs, leading to mortality (Fig. 2I–L). Greater than 80% of *Mm*‐infected nematodes died via bagging by 24 h post infection (Fig. S1). While both species of mycobacteria induce pathological changes*,* the immune response most likely clears *Ms*, preventing permanent damage, but cannot clear *Mm*.

### *Mm* colonize *C. elegans* more extensively than *Ms*

**Figure 3:**
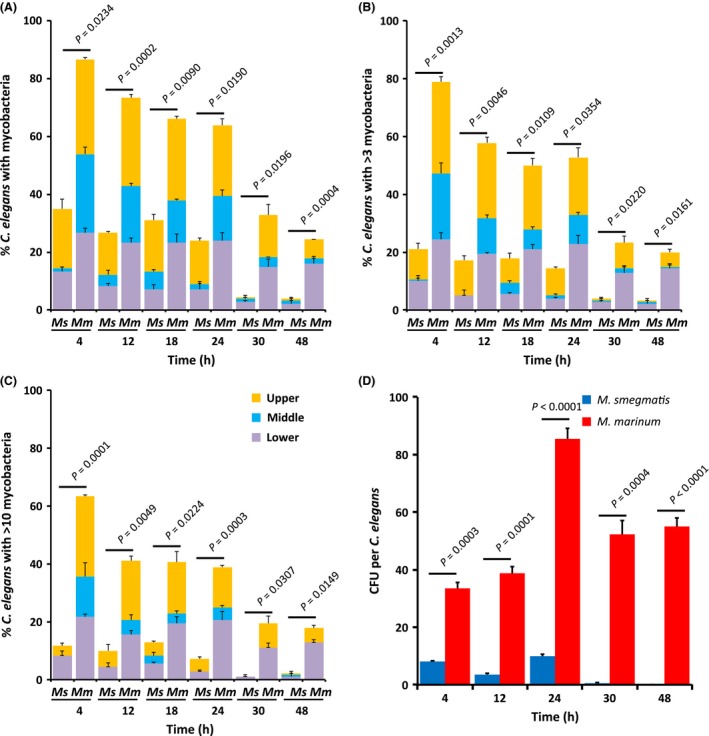
*M. marinum* colonize *C. elegans*. *C. elegans* were infected with either *M. smegmatis* (*Ms*) or *M. marinum* (*Mm*) for 24 h and quantified during and postinfection. 60 nematodes were randomly split into three groups of 20 at each time point. The number of bacteria in the upper, middle, and lower third of each nematode was determined. *P* ‐values at each time point are provided (unpaired *t*‐test). (A–C) Quantification of bacteria in the upper, middle, and lower regions of nematodes. Data are presented for means and standard deviations. Key in (C) is for (A–C). (A) *C. elegans* with at least one, (B) 4 or more, or (C) 11 or more cluster(s) of mycobacteria in the upper, middle, or lower segment. (D) Colony‐forming units (CFU) of mycobacteria per nematode recovered from 20 to 30°C. elegans at each time point.

We infected green fluorescent *C. elegans* with red fluorescent *Mm* (ψms91) and *Ms* (ψms23), and found that they behave similarly to their nonfluorescent counterparts (Fig. S3 and S4). These observations allowed use of fluorescent markers to localize the bacteria during infection. *Mm* and *Ms* are found within *C. elegans* 4 h during infection (Fig. 2 M and S). At 4 h, >60% of the nematodes are colonized by *Mm*, with >10 bacteria/nematode (Fig. 3C). In contrast, <15% of the nematodes display >10 intact *Ms* per nematode. Apparently, *C. elegans* can digest *Ms* in a manner similar to *E. coli*, but *Mm* persists. Quantitative analyses by microscopy confirm that *Ms* is present in lower quantities than *Mm* at all time points (Fig. 3). The majority of mycobacteria are within the pharyngeal and lower gut regions. Even 6 h after the 24 h infection period, *Ms* was barely observable within nematodes, while *Mm* persists (Figs. 2 and 3). We found that >50% of *C. elegans* carried *Mm*, while <5% still had *Ms* at 24 h post infection. We confirmed observations from fluorescent microscopy by plating for colony‐forming units (CFU). We consistently observed >2‐fold higher levels of *Mm* CFU in *C. elegans* as compared with *Ms* (Fig. 3D). We confirmed that the fluorescent labels in the bacteria do not impact these observations using unlabeled organisms in similar experiments and obtained comparable results (Fig. S5). There is a decrease in *Ms* within nematodes by 6 h post infection; whereas, *Mm* persists beyond 24 h post infection.

### *Mm* colonize the *C. elegans* intestine

**Figure 4:**
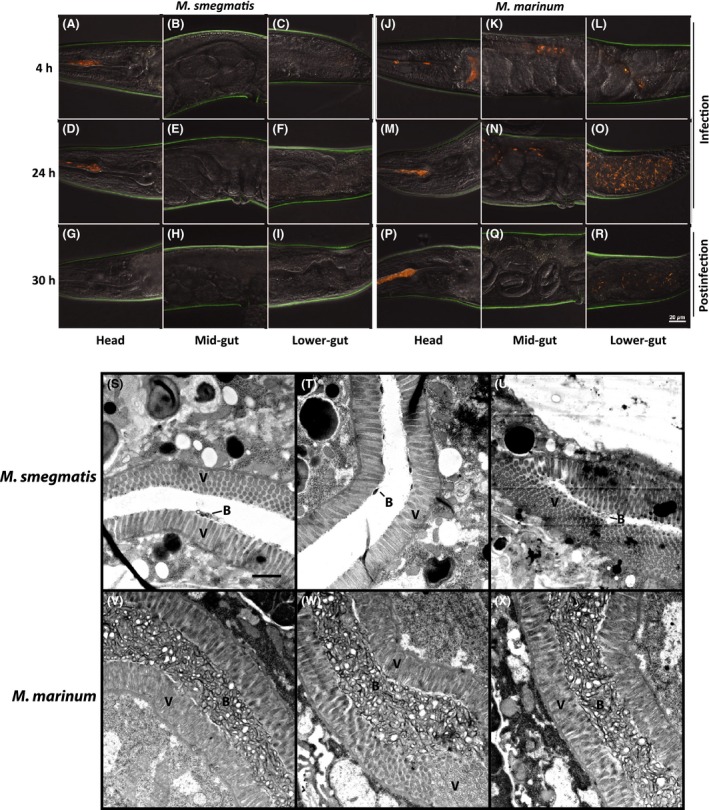
Confocal and transmission electron microscopy of infected *C. elegans*. *C. elegans* infected with (A–C, G–I, M–O, S–U) *M. smegmatis* (tdTomato) or (D–F, J–L, *P*–R, V–X) *M. marinum* (tdTomato) were imaged at (A–F) 4 and (G–L) 24 h during infection and (M–X) 6 h postinfection (30 h). (A–R) The head, mid‐gut, and lower‐gut of 13–15 nematodes each at 4, 24, and 30 h were imaged using a confocal microscope and (S–X) at 30 h using transmission electron microscopy. A spectral filter for excitation wavelengths of 500–640 nm was used for confocal microscopy. The scale bar in R (20 *μ*m) applies to panels A–R. The scale bar in S (1 *μ*m) applies to panels S–X. V indicates nematode villi and B indicate mycobacterial debris and mycobacteria.

In order to gain insight into the mechanisms involved, we imaged infected *C. elegans* using high‐resolution confocal and electron microscopy. We imaged the head, mid‐gut, and lower‐gut regions of nematodes where accumulations of mycobacteria were observed (Figs. 3 and 4). Bacteria are found in the head from 4 to 24 h during infection, but by 6 h post infection *Ms* is cleared and *Mm* is retained. *Ms* is rarely found in the lower gut, though some individual bacteria are observed. In contrast, *Mm* is present in the lower gut throughout and after the infection period. Observations by electron microscopy correlated well with those by confocal, with numerous *Mm* debris and bacteria present in the intestine; whereas, little *Ms* debris remains (Fig. 3 S–X). Interestingly, the villi of the *Mm*‐infected nematodes were less well organized and had greater variability in density, suggesting that *Mm* disrupts the actin core of villi, known to be composed of ACT‐5 (MacQueen et al. 2005).

### *C. elegans* is a novel virulence model for *Mm*

**Figure 5:**
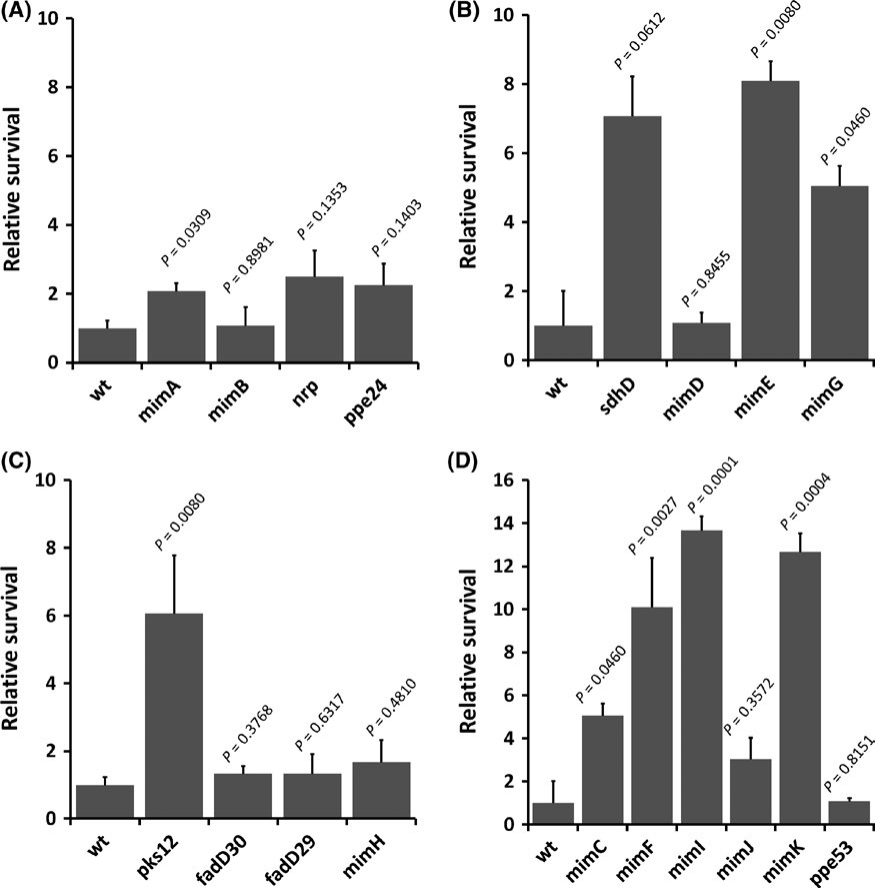
*C. elegans* can be used to measure virulence of *M. marinum*. (A–D) Relative survival of *C. elegans* infected with *M. marinum* wild type (wt) and macrophage infection mutants (MIMs), 2 d postinfection (day 6). Three trials of 20 nematodes (*n* = 60) were infected with each of the MIM strains and survival was assessed on day 6. All survival calculations are relative to survival after infection with wild type, e.g. relative survival = number of nematodes surviving with strain/number of nematodes surviving with wild type. Data are presented for means and standard deviations. *P* values are provided comparing relative survival with each MIM to infection with wild type (unpaired *t*‐test).

*Mm* mutants that are defective for infection and growth within mammalian macrophages (Mehta et al. 2006) were screened for pathogenesis in *C. elegans* (Fig. 5). A total of eight (44%) of the *Mm* mutants were attenuated in *C. elegans* (*P* < 0.05). These observations suggest that there are parallels between the *Mm* genes involved in mammalian macrophage infection and those required for colonization of *C. elegans*. Three of these mutants, *mimA*,* mimG*, and *mimI* were complemented with their respective gene, restoring wild‐type levels of *C. elegans* killing, confirming that these genes are specifically involved in *C. elegans* pathogenesis (Fig. S6). Our results suggest that *C. elegans* is a genetically tractable host for study of *Mm* pathogenesis and can be used to analyze virulence mechanisms in a manner that complements existing mammalian models.

### p38 MAPK plays an important role in protection

**Figure 6:**
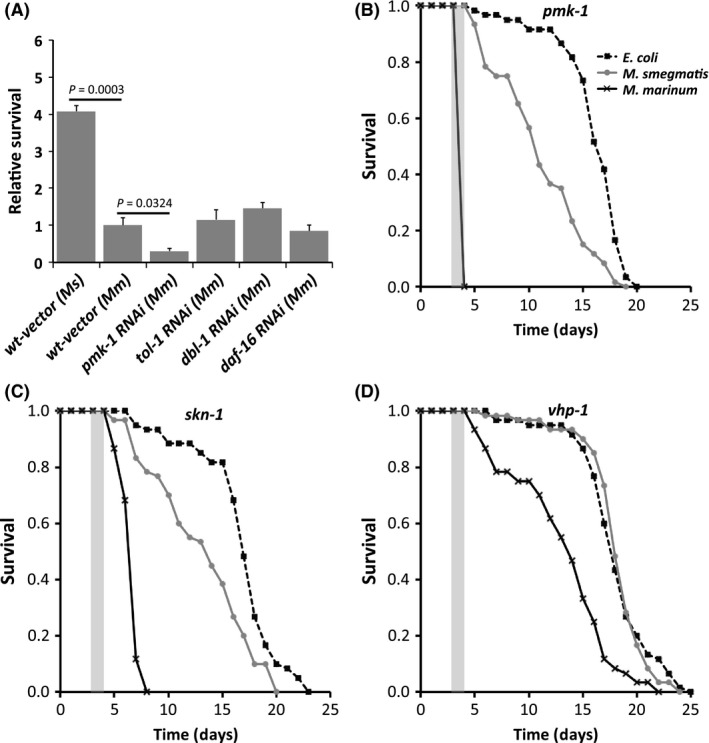
Pathogenic mycobacteria block *C. elegans* MAPK‐mediated protection using MAPK phosphatase. (A) Relative survival of RNAi knock‐down of p38 MAPK (*pmk‐1*) TLR (*tol‐1*), TGF‐ β (*dbl‐1*) and insulin‐like receptor (*daf‐16*) at 2 d postinfection as compared with vector alone wild type. Relative survival = number of RNAi knock‐down nematodes surviving/number of wild‐type surviving. Data are means and standard deviations. (B) Survival curve for MAPK (*pmk*‐1) mutant after 24 h of infection. *Mm* versus *Ms P* < 0.0001; *Mm* versus *E. coli P* < 0.0001; *Ms* versus *E. coli P* < 0.0001. Key in (B) is for (B‐D). (C) Survival curve for the *C. elegans* the downstream regulator of MAPK (skn‐1) mutant after 24 h infection. *Mm* versus *Ms P* < 0.0001; *Mm* versus *E. coli P* < 0.0001; *Ms* versus *E. coli* *P* < 0.0001. (D) Survival curve for the *C. elegans* MAPK phosphatase (vhp‐1) mutant after 24 h infection. *Mm* versus *Ms P* < 0.0001; *Mm* versus *E. coli P* < 0.0001; *Ms* versus *E. coli P* = 0.9173. Survival curve *P* values from log‐rank analysis.

The p38 MAPK pathway is triggered during the initial response of mammalian macrophages to mycobacterial infection (Roach and Schorey 2002), but its role in the multi‐cellular response in whole animals has been difficult to determine due to the essential nature of this gene during mammalian development. The p38 MAPK can play a role in protection of *C. elegans* from other pathogens, but mycobacteria have not been examined (Aballay et al. 2003; Jebamercy et al. 2013). We used *E. coli* expressing RNAi to knock‐down p38 MAPK (*pmk‐1*) expression as well as other relevant genes in *C. elegans*. We confirmed that all RNAi knock‐downs display decreased expression of the targeted gene by qPCR. There was >10‐fold decrease in expression for all genes, other than *vhp‐1* (MAPK phosphatase), which displayed a >4‐fold decrease. *C. elegans* with reduced MAPK expression are more susceptible to both *Mm* and *Ms* (Fig. 6A, S7). We confirmed that this phenotype was not due to off‐target effects of RNAi using a specific *C. elegans* p38 MAPK (*pmk‐1)* mutant. The p38 MAPK mutant displays a similar increase in mortality, with 100% mortality during the first 24 h (Fig. 6B, S7). The *C. elegans* MAPK mutant not only displayed an increase in mortality, but also increased pathology (Fig. S8), demonstrating that p38 MAPK is an important pathway for protection from mycobacteria.

### MAPK is the dominant pathway involved in protection from mycobacteria

Several alternative signaling pathways are important in defense of *C. elegans* from other pathogenic bacteria, including TLR, TGF‐*β*, and insulin‐like receptor pathways (Evans et al. 2008; Tenor and Aballay 2008; Singh and Aballay 2009). We used RNAi to decrease expression of *tol‐1* (TLR), *dbl‐1* (TGF‐*β* pathway), and *daf‐16* (insulin‐like pathway) genes in *C. elegans* and evaluated mycobacterial susceptibility. None of these signaling pathways had an impact on susceptibility (Fig. 6A). We confirmed these results using *C. elegans* mutants in *tol‐1, dbl‐1*, and *daf‐16* (Fig. S9 and also found no impact on susceptibility. In fact, *C. elegans* mutants in other signaling pathways display a trend toward increased resistance to mycobacterial infection. As expected, the *C. elegans tol‐1, dbl‐1*, and *daf‐16* mutants display similar pathology to wild‐type infected with *Ms* and *Mm* (Fig. S10, S11 and S12). These observations suggest that the *C. elegans* model for mycobacterial infection has the advantage that it can be used to focus analysis of host protection and susceptibility mechanisms on the p38 MAPK pathway.

### MAPK mediates protection through SKN‐1

One of the primary downstream regulators that *C. elegans* p38 MAPK controls is SKN‐1 (Papp et al. 2012). The *skn‐1* gene, analogous to the essential mammalian *nrf1* and *nrf2* cap’n’collar subfamily of basic leucine zipper transcription factors, is activated during the *C. elegans* response to oxidative stress and bacterial infection (Papp et al. 2012). We infected *C. elegans skn‐1* RNAi knock‐down nematodes and found no difference compared with wild type in susceptibility as measured by survival (Fig. S13). However, we found that *Mm*‐infected *skn‐1* RNAi knock‐down *C. elegans* display increased depigmentation and shortened length, suggesting that the role of *skn‐1* is only partially observed in knock‐down strains. We investigated the possibility that a complete *skn‐1* knockout would have a more obvious phenotype using a *skn‐1* mutant. The *skn‐1* mutant displays more rapid mortality as compared with wild type after infection with *Mm* (Fig. 6C). The *Mm*‐infected *skn‐1* mutant displays depigmentation, shortening, and bagging (Fig. S13), but less bagging at 48 h than the *pmk‐1* mutant (*P* < 0.0001), demonstrating that SKN‐1 is a downstream regulator for MAPK‐mediated protection against mycobacteria in *C. elegans*.

### Hijacking the host mapk phosphatase

MAPK phosphatase (*vhp‐1)* acts as an inhibitor of the p38 MAPK pathway and can modulate the innate immune response (Kim et al. 2004). We evaluated the role of MAPK phosphatase in the susceptibility of *C. elegans* to mycobacterial infection using RNAi. We found that the *C. elegans vhp‐1* knock‐down strain displayed increased survival after infection with *Mm* (Fig. S15). Similarly, a *vhp‐1* mutant displayed improved survival postinfection by *Mm* (Fig. 6D). The *vhp‐1* mutant also displays a reduction in bagging (<5%) as compared with wild type (>80%) and the *pmk‐1* mutant (100%) (Fig. S16), demonstrating that the p38 MAPK pathway is involved in combating mycobacterial infection and that the presence of *vhp‐1* increases susceptibility to mycobacteria in *C. elegans*.

## Discussion

The *C. elegans* model provides an extremely simple and effective system for pathogenesis studies, particularly mechanisms for avoiding ROS and lysosomal defenses (Mallo et al. 2002; Chavez et al. 2007). Research has focused on the adaptive immune response to pathogenic mycobacteria, but the innate immune response and roles of MAPK are not fully understood (Philips and Ernst 2012). Part of the reason that *C. elegans* has not been used to study *Mm* pathogenesis is because another study did not observe mortality (Couillault and Ewbank 2002), but conditions were not optimized. We used age‐synchronized, 3‐day‐old gravid adult nematodes and all infections were carried out at ~20°C. Use of adult gravid nematodes is important, since bagging and depigmentation are only observed in adults. The pathological processes of depigmentation and bagging are consistent with observations with other pathogens (Mosser et al. 2011). *Pseudomonas aeruginosa* was one of the first human pathogens studied in *C. elegans*, where they also cause increased mortality (Mahajan‐Miklos et al. 1999). *S. pyogenes, E. faecium*, and *P. aeruginosa* cause mortality via toxin‐mediated killing (Jansen et al. 2002; Moy et al. 2004). *E. faecalis, S. marcescens*, and *S. enterica* attach to the gut epithelium of *C. elegans* (Kurz et al. 2003; Maadani et al. 2007; Sem and Rhen 2012). The actinomycete *Streptomyces albireticuli* and the fungus *Drechmeria coniospora,* natural pathogens of *C. elegans*, invade the gut epithelium (Jansson et al. 1984; Park et al. 2002). Interestingly, *Microbacterium nematophilum* forms an adhesive biofilm on the *C. elegans* cuticle (Hodgkin et al. 2000). Despite the diversity of bacterial interactions observed, the mechanisms of susceptibility to mycobacteria are relatively unique.

*Mm* cause mortality in *C. elegans* through inhibition of the nematode's ability to lay eggs. Inhibition of egg laying causes infected adult nematodes to produce a bag of nematodes (bagging), which is protection for the progeny (Mosser et al. 2011). Most likely the bagging phenotype is the result of stress induced within the worm as a result of attempts to defend against mycobacterial invasion of the intestinal epithelium, involving production of reactive oxygen species. Increased oxidative stress causes loss of pigmentation in adult nematodes and lower survival. Future studies evaluating the mechanisms of *Mm* pathogenesis in the absence of bagging would be an important next step using this host–pathogen model. These studies could be accomplished with *C. elegans* mutants that do not produce progeny, CF512: *fer‐15;fem‐1*, and thereby, would not die due to bagging (Troemel et al. 2006). The identification of several Mm mutants that impact mortality due to bagging suggests that specific Mm pathogenic mechanisms are involved and separation from early mortality due to bagging might offer one way to gain further discriminatory power for subtle virulence defects. Interestingly, we also observed differences in time to death and percent mortality when worms are exposed to more *M. marinum*, as shown in Figure 1. These observations suggest that additional discriminatory power for subtle virulence defects might be accomplished by variation in the exposure time to *M. marinum* mutants in different virulence pathways of interest.

On the surface, one might consider the absence of a clear macrophage lineage of cells in *C. elegans* (Engelmann and Pujol 2010) to be a disadvantage for study of intracellular pathogens, including *Mm*. However, other intracellular pathogens, including *Legionella pneumophila* (Komura et al. 2010), have been successfully examined using *C. elegans* as a host that allowed analysis of specific virulence mechanisms playing an important role within macrophages. There are specialized cells that continuously endocytose fluid from the lumen of the worm intestine called coelomocytes, but these have not been shown to phagocytose large particles (Fares and Greenwald 2001). Interestingly, when ROS and lysozymes are produced in worms, these bactericidal host defense molecules are delivered to the lumen of the worm intestine (Mallo et al. 2002; Chavez et al. 2007), rather than to an endosomal or sorting compartment, as would occur within macrophages or single‐cell phagocytes, such as amoebae (Cirillo 1999; Yan et al. 2004). These observations, combined with those within this study, suggest the interesting concept that, in the case of *C. elegans*, the lumen of the worm intestine might serve a similar role to the sorting compartment where food or energy sources are sorted from toxic or pathogenic particles and pathogenic particles are attacked with ROS, lysozyme, and potentially other innate immune effectors to defend the worm. Thus, the entire digestive tract of the worm serves as an analogous system to that of a phagocyte. Using this analogy, the worm phagocytoses bacteria by eating them and the lumen of the worm intestine serves as an endosome, with which ROS and lysosomal vesicles fuse, resulting in bacterial killing and degradation for nonpathogenic species, but pathogens resist or inhibit these innate immune mechanisms.

**Figure 7:**
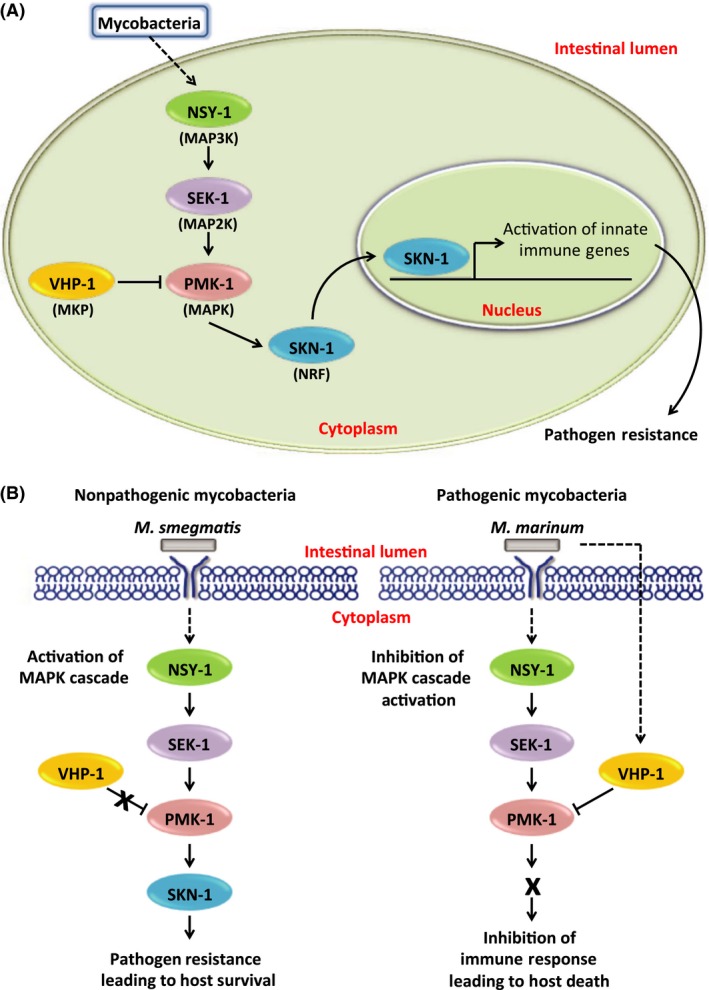
Mycobacterial mechanism of MAPK pathway control during *C. elegans* infection. (A) When *C. elegans* is infected with mycobacteria, the p38 MAPK pathway (PMK‐1) is activated, leading to an innate immune response through downstream regulators, including the NRF‐like regulator SKN‐1, that increase pathogen resistance. The MAPK phosphatase, VHP‐1, inhibits activation of MAPK, but is inactivated when foreign organisms are recognized, allowing MAPK‐mediated protection. (B) In contrast to nonpathogenic mycobacteria (*M. smegmatis*), pathogenic mycobacteria (*M. marinum*) may inhibit activation of MAPK through the MAPK phosphatase VHP‐1, allowing colonization and pathogenesis.

Activation of MAPK differs between pathogenic and nonpathogenic mycobacteria infection in *C. elegans*, consistent with observations in mammalian tissue culture models (Roach and Schorey 2002; Schorey and Cooper 2003). The p38 MAPK plays a role in protection, but MAPK phosphatase (*vhp‐1*) plays a role in susceptibility*. C. elegans* lacking p38 MAPK, display 100% mortality, which is reduced in the absence of *vhp‐1. Ms* causes comparable mortality rates to *E. coli* in the *vhp‐1* mutant, similar to the *tol‐1*,* dbl‐1*, or *daf‐16* mutants. Thus, susceptibility to *Mm* primarily involves MAPK. While the *skn‐1* mutant does not display the same bagging phenotype as the MAPK mutant, it displays increased depigmentation and reduced survival. Although these observations demonstrate the importance of *skn‐1* in protection from mycobacteria, the more modest phenotype suggests that there are additional downstream mediators of MAPK protection yet to be identified. Further studies could identify additional downstream regulatory genes and the effectors involved in MAPK‐mediated protection from mycobacteria. Apparently, *C. elegans* responds differently to pathogenic and nonpathogenic mycobacteria through different levels of MAPK activation (Fig. 7). Nonpathogenic mycobacteria activate MAPK leading to bacterial killing and *C. elegans* survival, but pathogenic mycobacteria may utilize *vhp‐1* to reduce activation of MAPK*,* leading to susceptibility and mortality. Similarly, mycobacterial virulence inversely correlates with levels of MAPK activation in mammalian macrophages (Roach and Schorey 2002; Schorey and Cooper 2003). The mechanisms involved in this differential activation of MAPK were previously unknown, demonstrating that analysis of interactions of pathogenic mycobacteria with *C. elegans* can provide insight into mechanistic differences that lead to pathogenesis. Further study of *vhp‐1* in *C. elegans* is needed, since it is likely that its absence also affects other kinases that play a role in host defense.

The ability of *Mm* to colonize *C. elegans* is due to interactions with the intestine, allowing elucidation of the bacterial ligands and receptors involved. We identified eight *Mm* mutants that are defective for infection of mammalian macrophages as well as causing less mortality in *C. elegans*, suggesting the mechanisms are relevant to mammalian pathogenesis. Colonization of the *C. elegans* intestine produces morphological changes in actin within villi, consistent with known interactions of pathogenic mycobacteria with actin during infection of macrophages (Guerin and de Chastellier 2000; Anes et al. 2003), suggesting that *C. elegans* can be used for study of mycobacterial molecular pathogenesis by allowing analysis of signaling pathways involved in innate immunity, mechanisms of resistance to ROS and lysosomes, and mechanisms of colonization and remodeling of actin. The tractability of *C. elegans* will facilitate mechanistic analysis of both the protective response to mycobacteria and virulence pathways, providing insight into novel strategies to prevent pathogenesis.

## Conflicts of Interest

No authors have a conflict of interest.

## Supporting information


**Figure S1.** Pathological changes in wild‐type (N2) *C. elegans* infected with bacteria.

**Figure S2.** Morphological characteristics of *C. elegans* (TP12) infected with *E. coli* (OP50).

**Figure S3.** Survival of TP12 after bacterial infection.

**Figure S4.** Pathological changes in TP12 *C. elegans* infected with bacteria.

**Figure S5.** Bacterial load in *C. elegans* (N2) determined by plating for CFU.

**Figure S6** ***.** C. elegans* Infected with complemented MIM of *M. marinum*.

**Figure S7.** Impact of C. elegans *pmk‐1* on infection with mycobacteria.

**Figure S8.** Pathological changes in *pmk‐1* Mutant *C. elegans* infected with bacteria.

**Figure S9.** The *C. elegans* tol‐1, dbl‐1, and daf‐16 Pathways Do Not Impact Mycobacterial Infection.

**Figure S10.** Pathological changes in *tol‐1* Mutant *C. elegans* infected with bacteria.

**Figure S11.** Pathological changes in *dbl‐1* Mutant *C. elegans* infected with bacteria.

**Figure S12.** Pathological changes in *daf‐16* Mutant *C. elegans* infected with bacteria.

**Figure S13.** Role of *C. elegans skn‐1* in mycobacterial infection.

**Figure S14.** Pathological changes in *skn‐1* Mutant *C. elegans* infected with bacteria

**Figure S15.** Role of *C. elegans vhp‐1* in mycobacterial infection.

**Figure S16.** Pathological changes in *vhp‐1* Mutant *C. elegans* infected with bacteria.

**Table S1.** Macrophage infection mutants (MIMs) of *M. marinum*.
**Table S2.** List of *C. elegans* RNAi constructs and mutant strains.
**Table S3.** Primers used for confirmation of *C. elegans* mutants and RNAi knock‐down.
