# Supplementary figures and images for: The Caenorhabditis elegans p38 MAPK Gene plays a key role in protection from mycobacteria
Source: Microbiologyopen. 2016 Feb 25;5(3):436–52. doi: 10.1002/mbo3.341 (PMC4905996; doi:10.1002/mbo3.341)

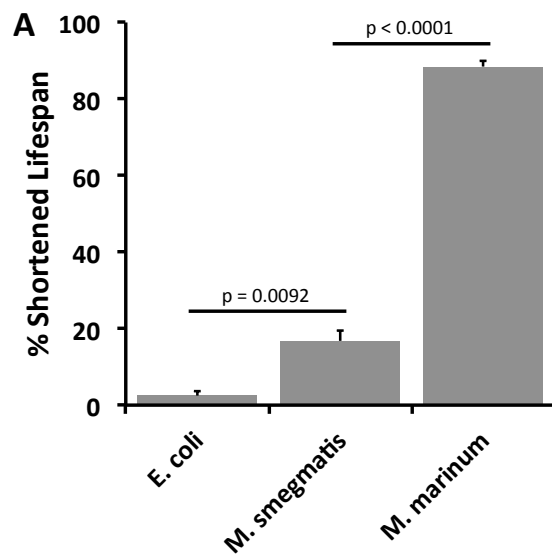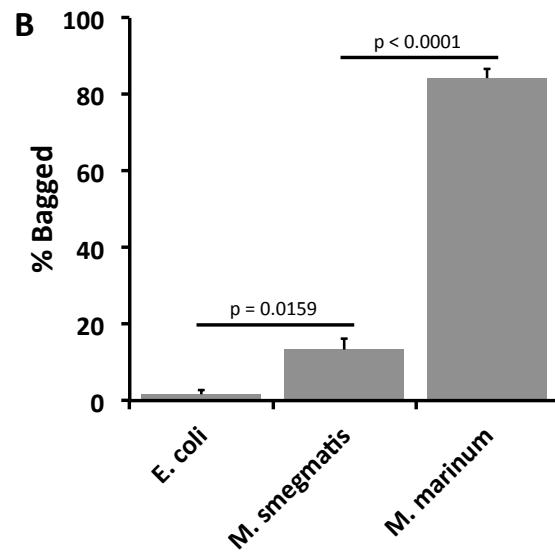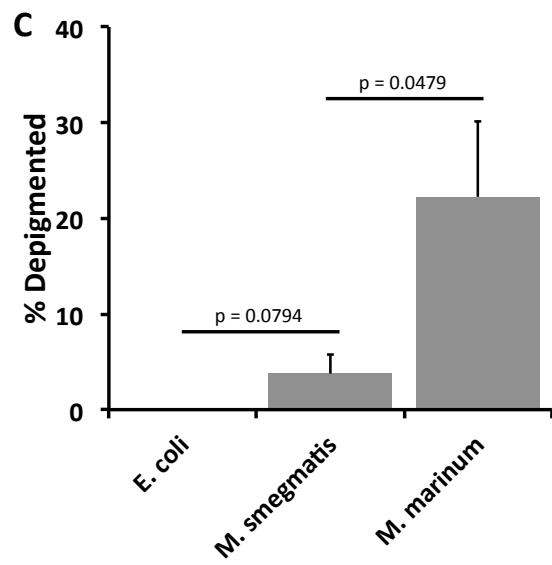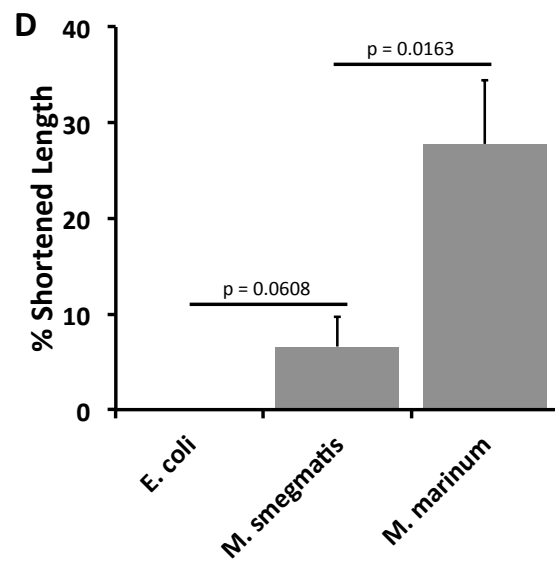

Supplement: Supplementary file 1 — Figure S1. Pathological changes in wild‐type (N2) C. elegans infected with bacteria. [file MBO3-5-436-s001.pdf]

*E. coli*

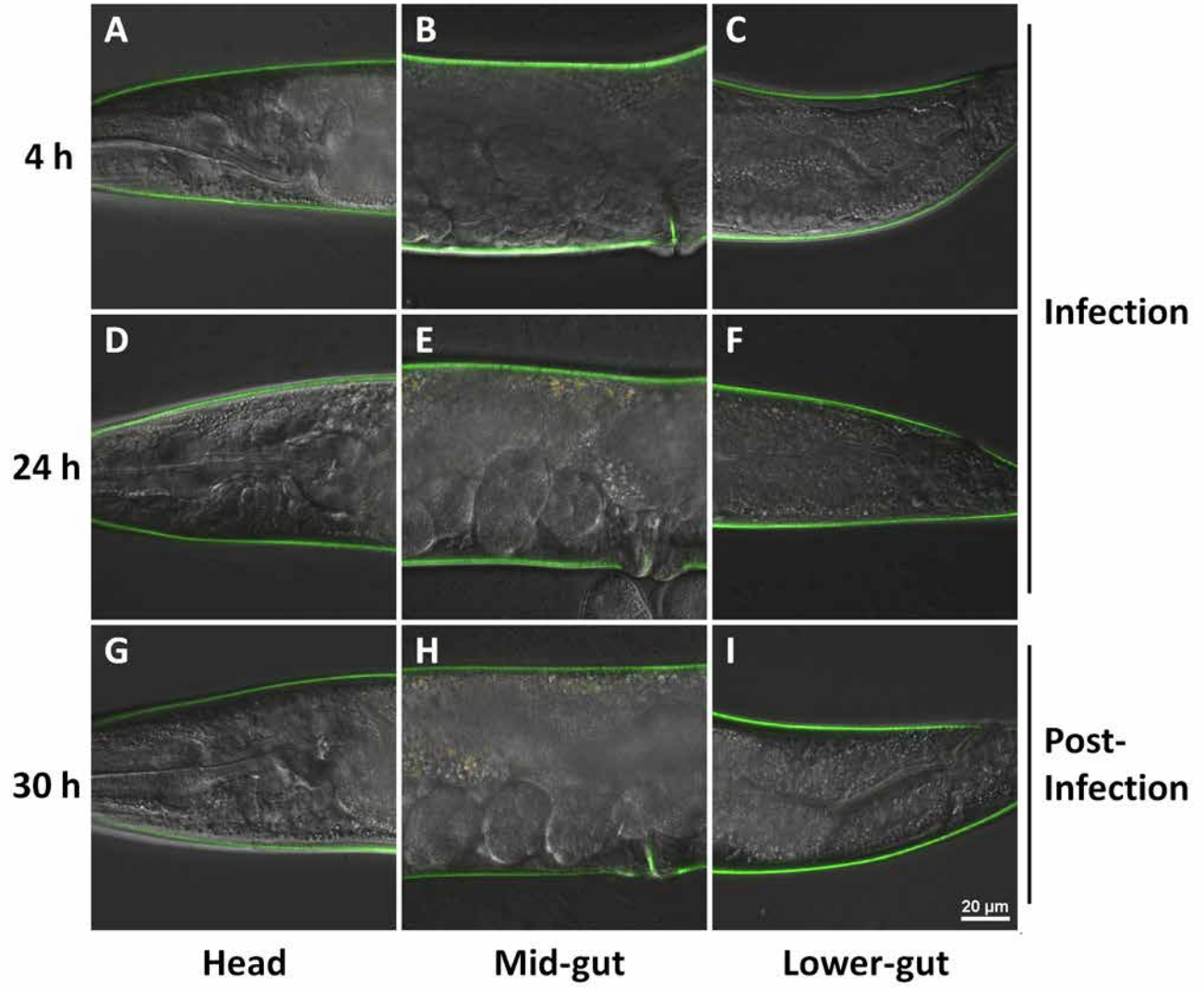

Supplement: Supplementary file 2 — Figure S2. Morphological characteristics of C. elegans (TP12) infected with E. coli (OP50). [file MBO3-5-436-s002.pdf]

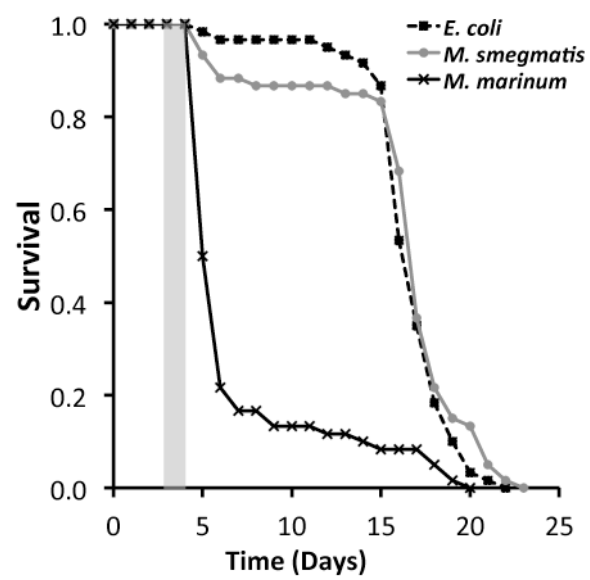

Supplement: Supplementary file 3 — Figure S3. Survival of TP12 after bacterial infection. [file MBO3-5-436-s003.pdf]

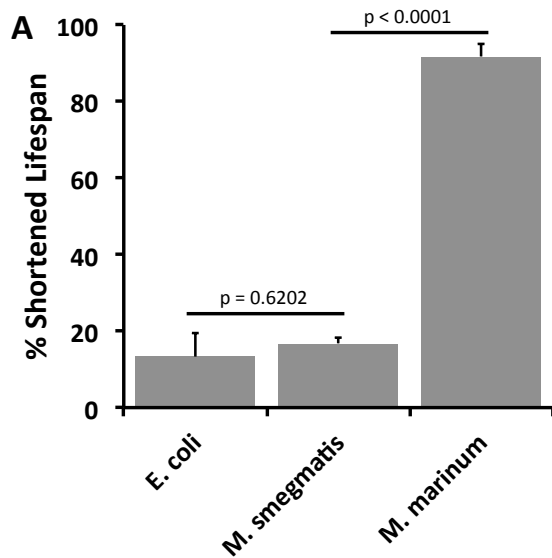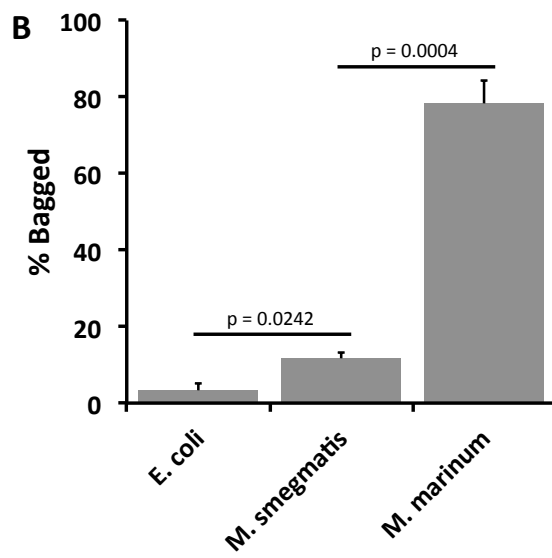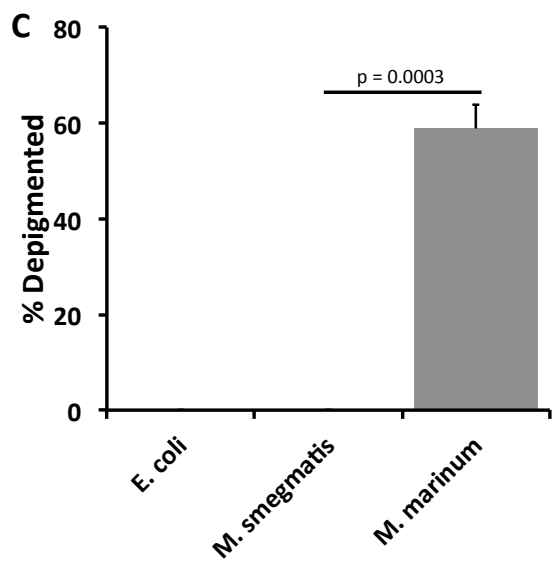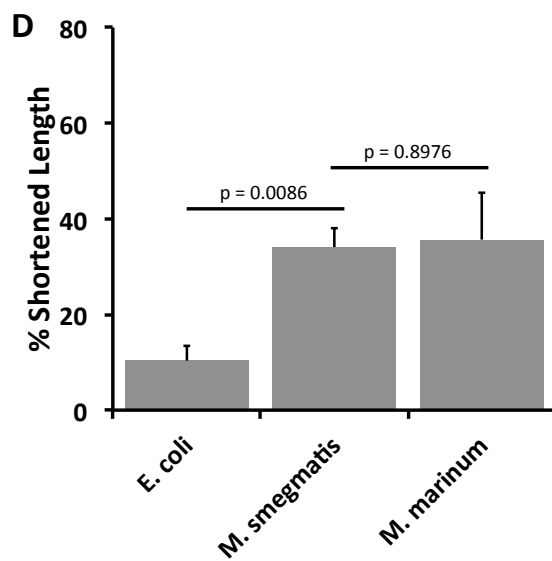

Supplement: Supplementary file 4 — Figure S4. Pathological changes in TP12 C. elegans infected with bacteria. [file MBO3-5-436-s004.pdf]

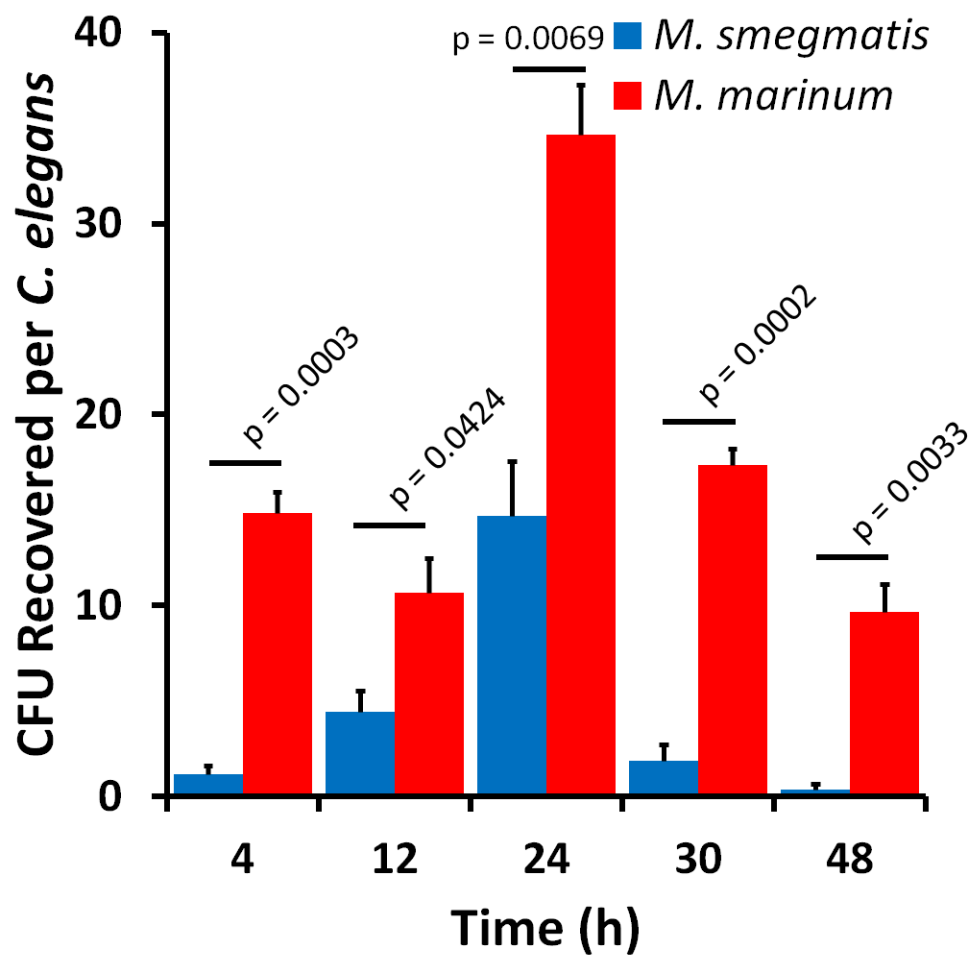

Supplement: Supplementary file 5 — Figure S5. Bacterial load in C. elegans (N2) determined by plating for CFU. [file MBO3-5-436-s005.pdf]

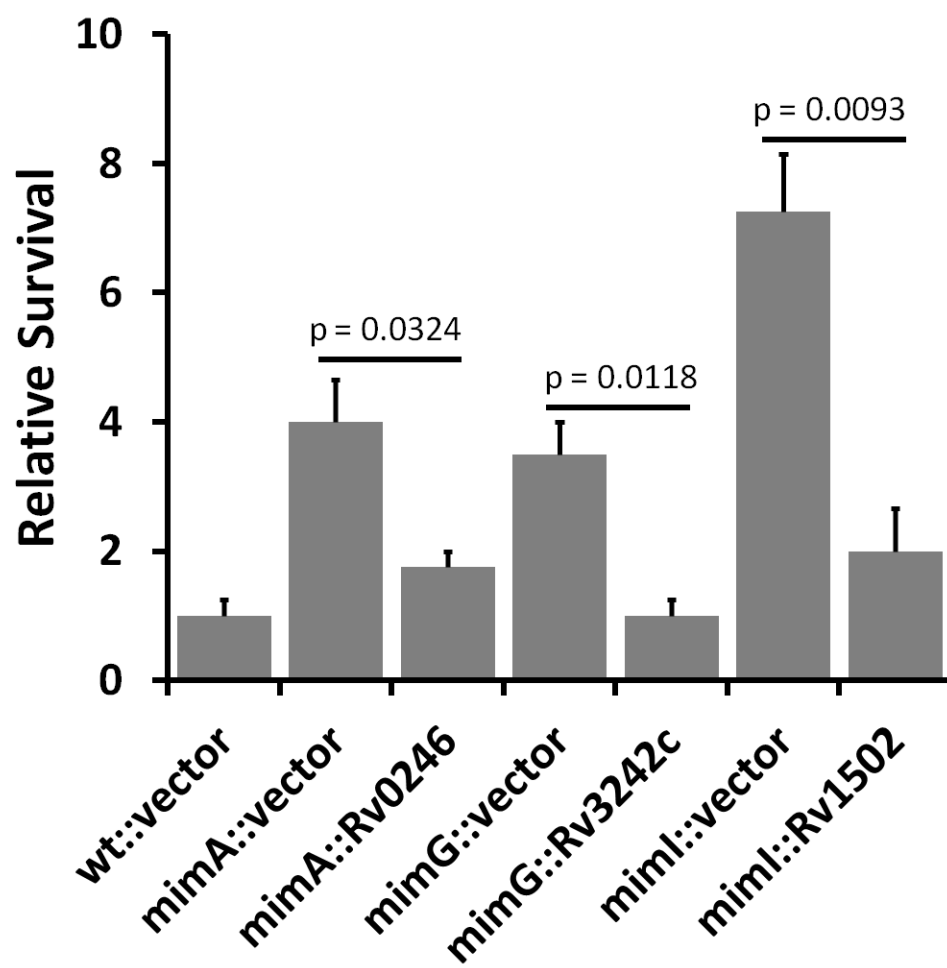

Supplement: Supplementary file 6 — Figure S6 . C. elegans Infected with complemented MIM of M. marinum. [file MBO3-5-436-s006.pdf]

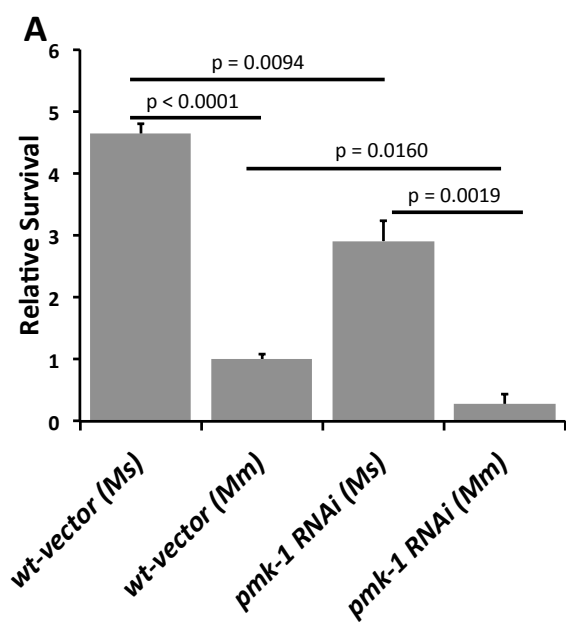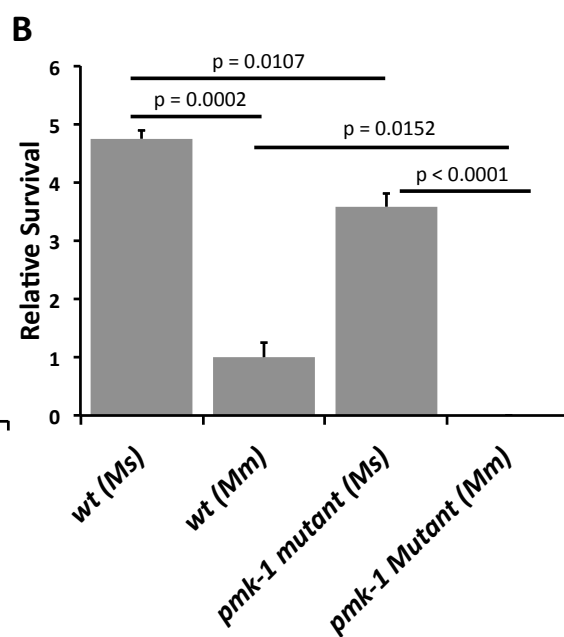

Supplement: Supplementary file 7 — Figure S7. Impact of C. elegans pmk‐1 on infection with mycobacteria. [file MBO3-5-436-s007.pdf]

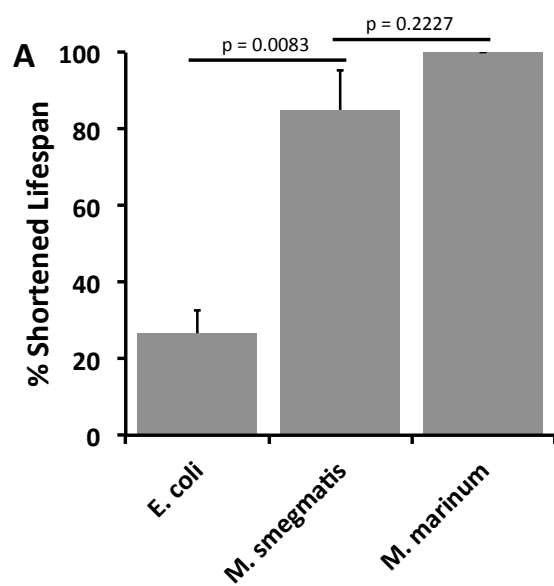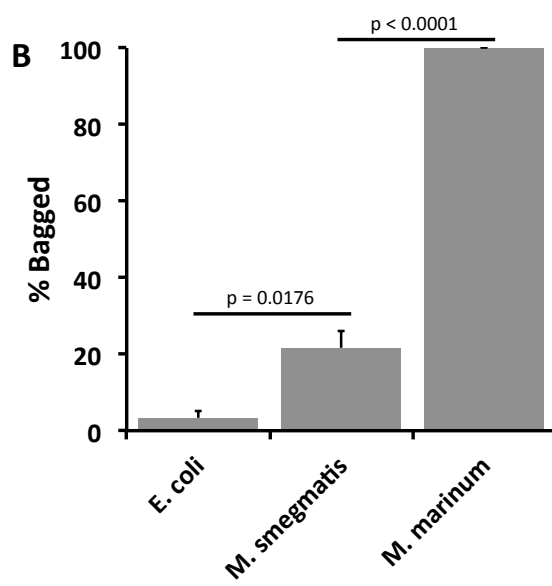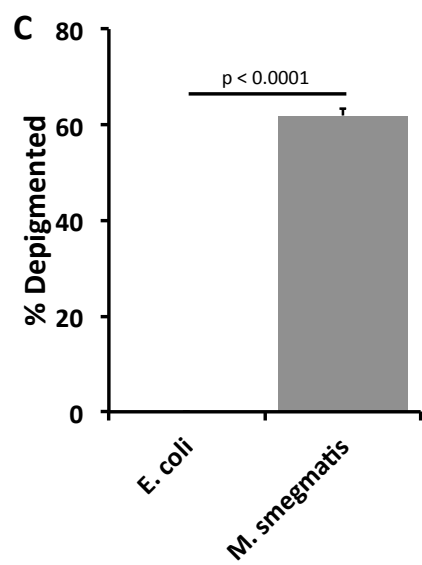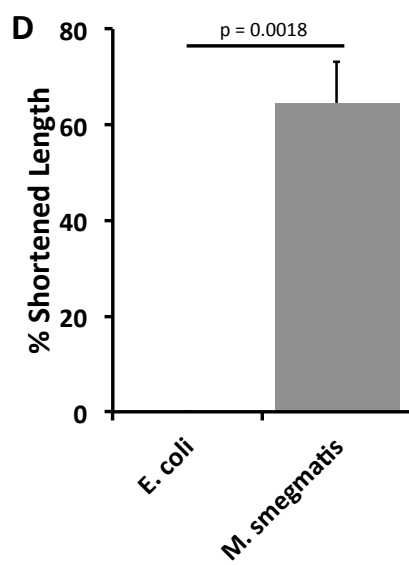

Supplement: Supplementary file 8 — Figure S8. Pathological changes in pmk‐1 Mutant C. elegans infected with bacteria. [file MBO3-5-436-s008.pdf]

**A**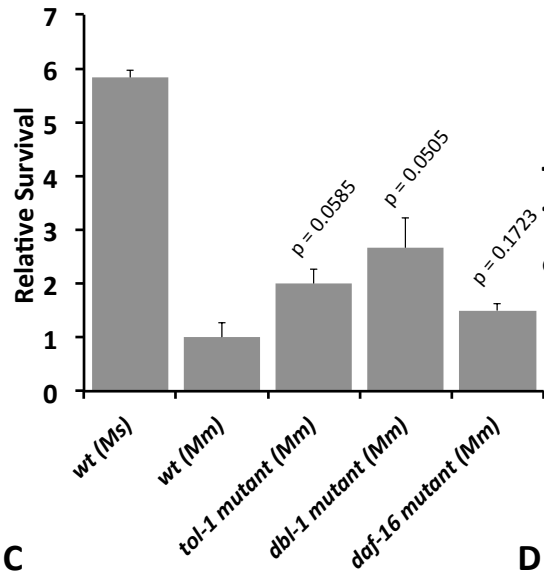**B**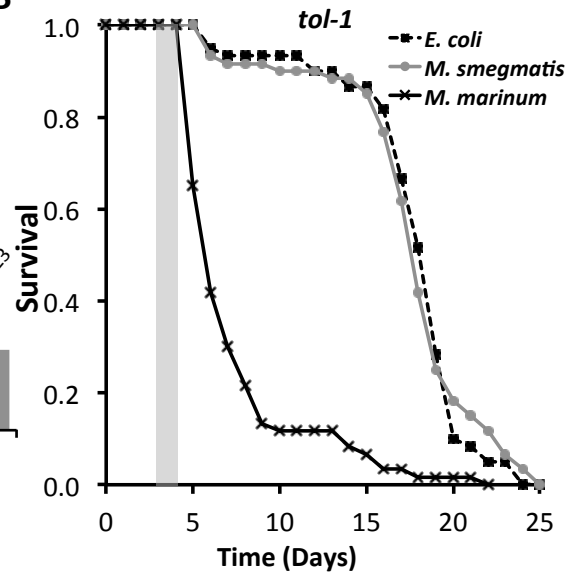**C**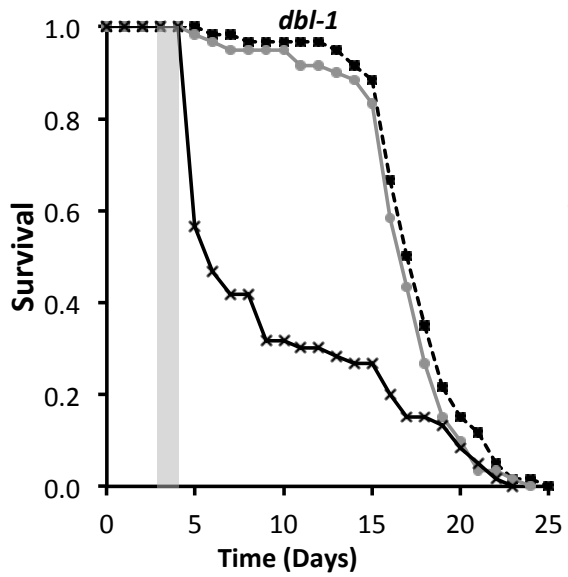**D**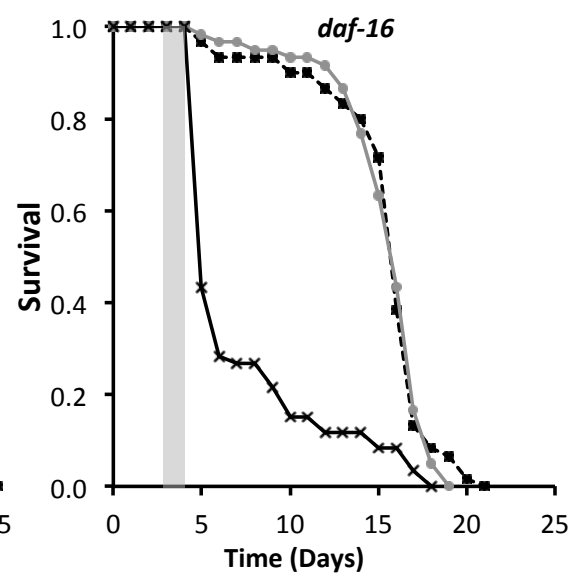

Supplement: Supplementary file 9 — Figure S9. The C. elegans tol‐1, dbl‐1, and daf‐16 Pathways Do Not Impact Mycobacterial Infection. [file MBO3-5-436-s009.pdf]

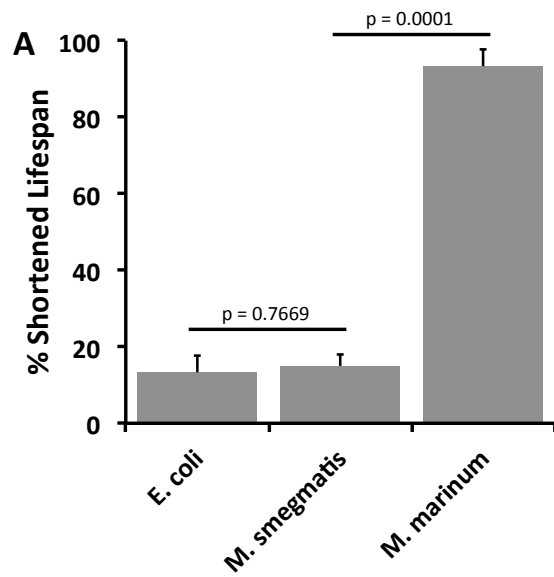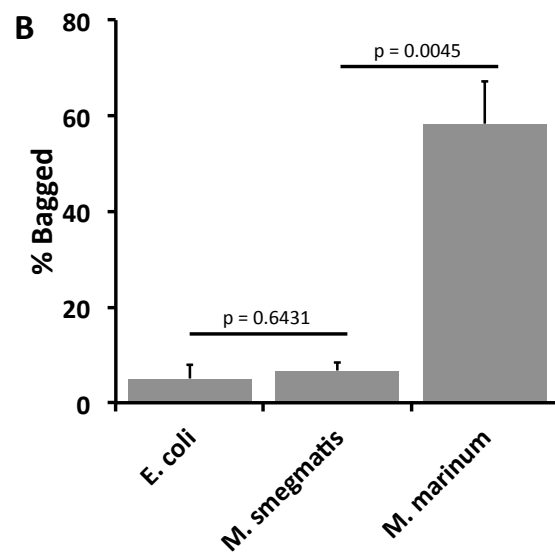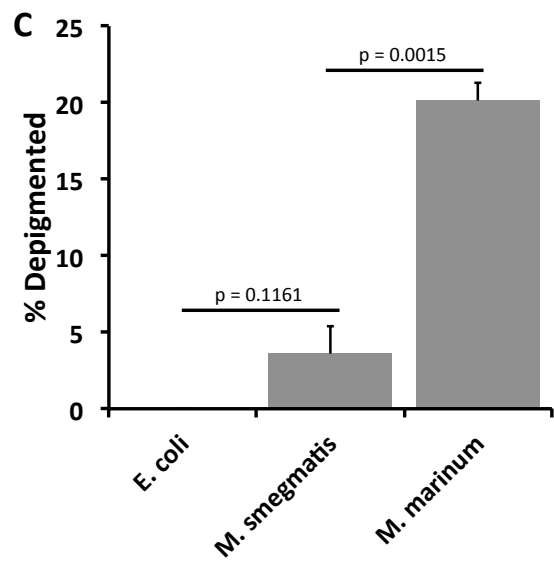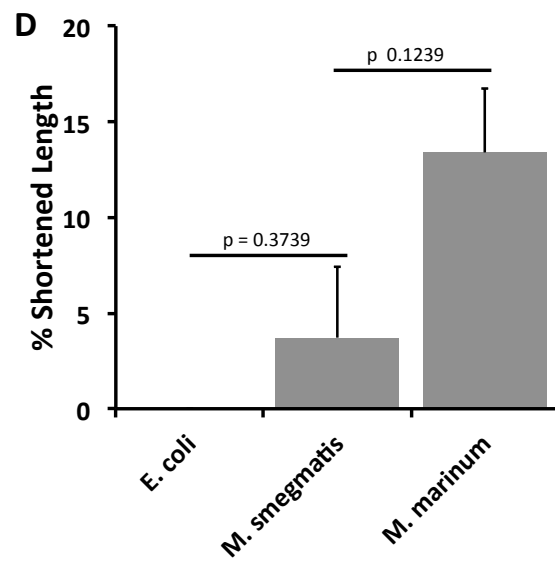

Supplement: Supplementary file 10 — Figure S10. Pathological changes in tol‐1 Mutant C. elegans infected with bacteria. [file MBO3-5-436-s010.pdf]

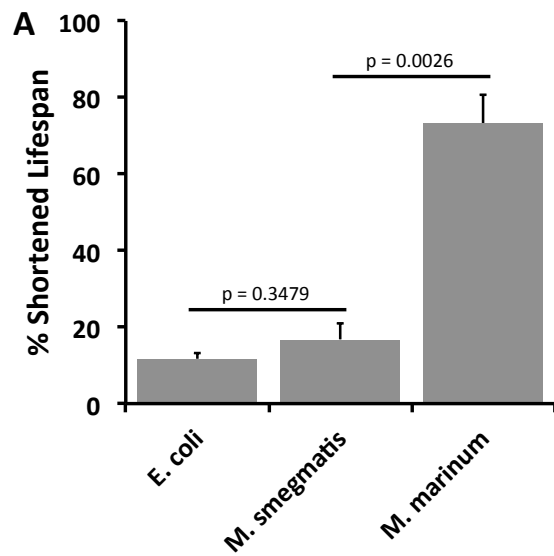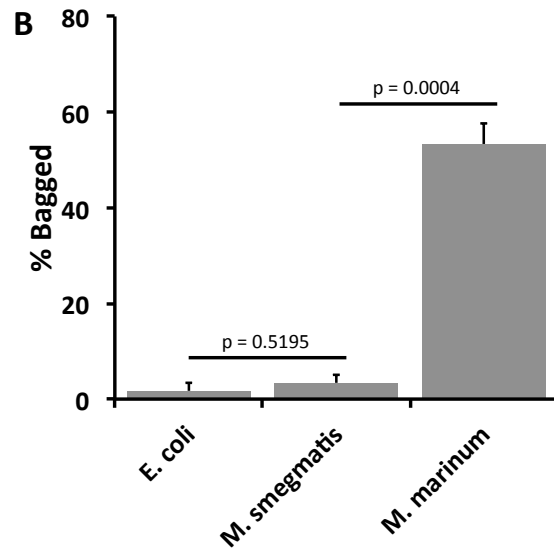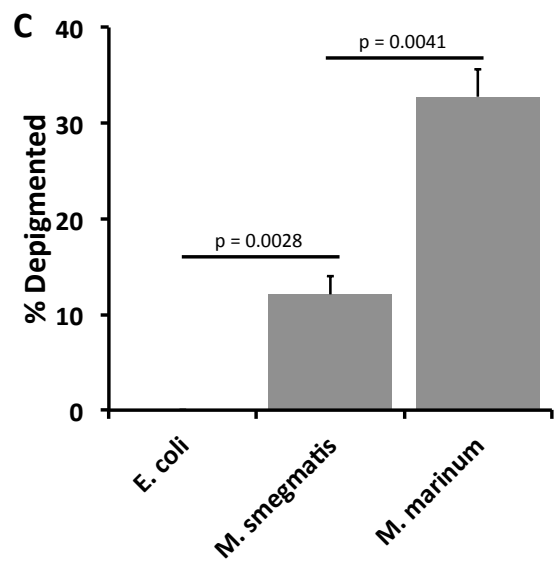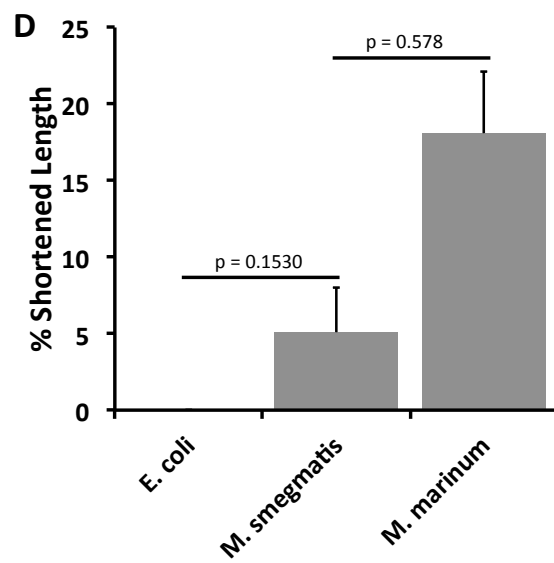

Supplement: Supplementary file 11 — Figure S11. Pathological changes in dbl‐1 Mutant C. elegans infected with bacteria. [file MBO3-5-436-s011.pdf]

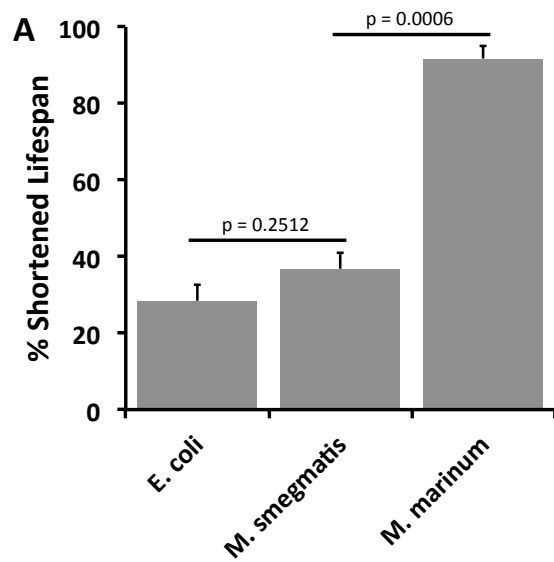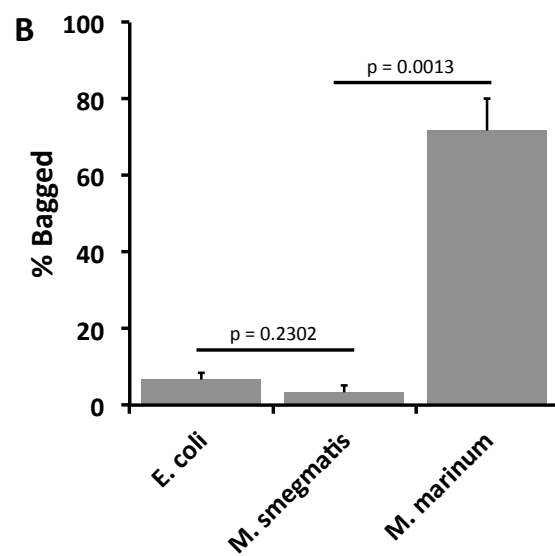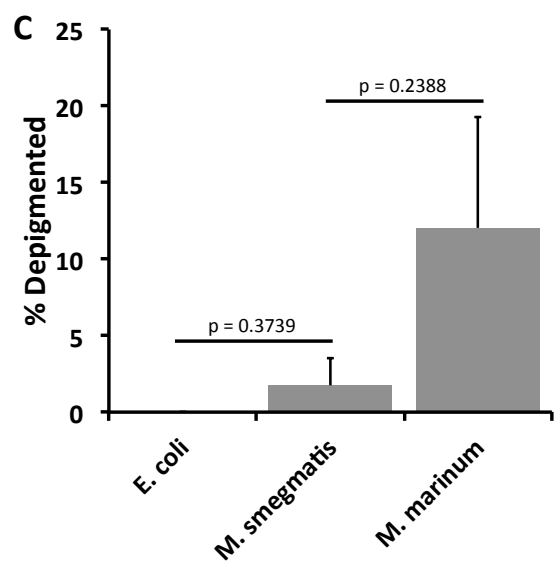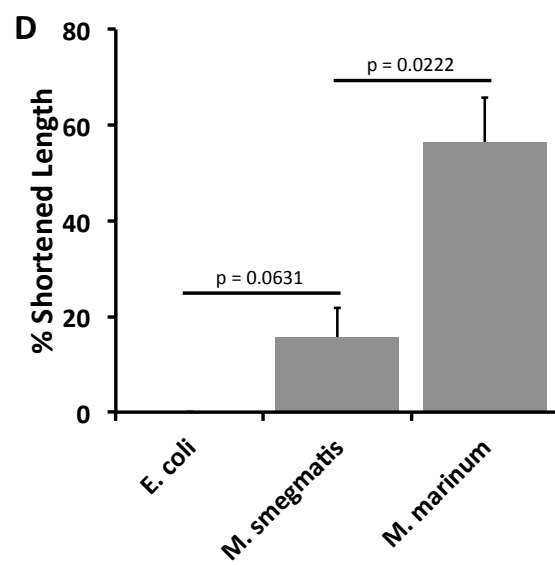

Supplement: Supplementary file 12 — Figure S12. Pathological changes in daf‐16 Mutant C. elegans infected with bacteria. [file MBO3-5-436-s012.pdf]

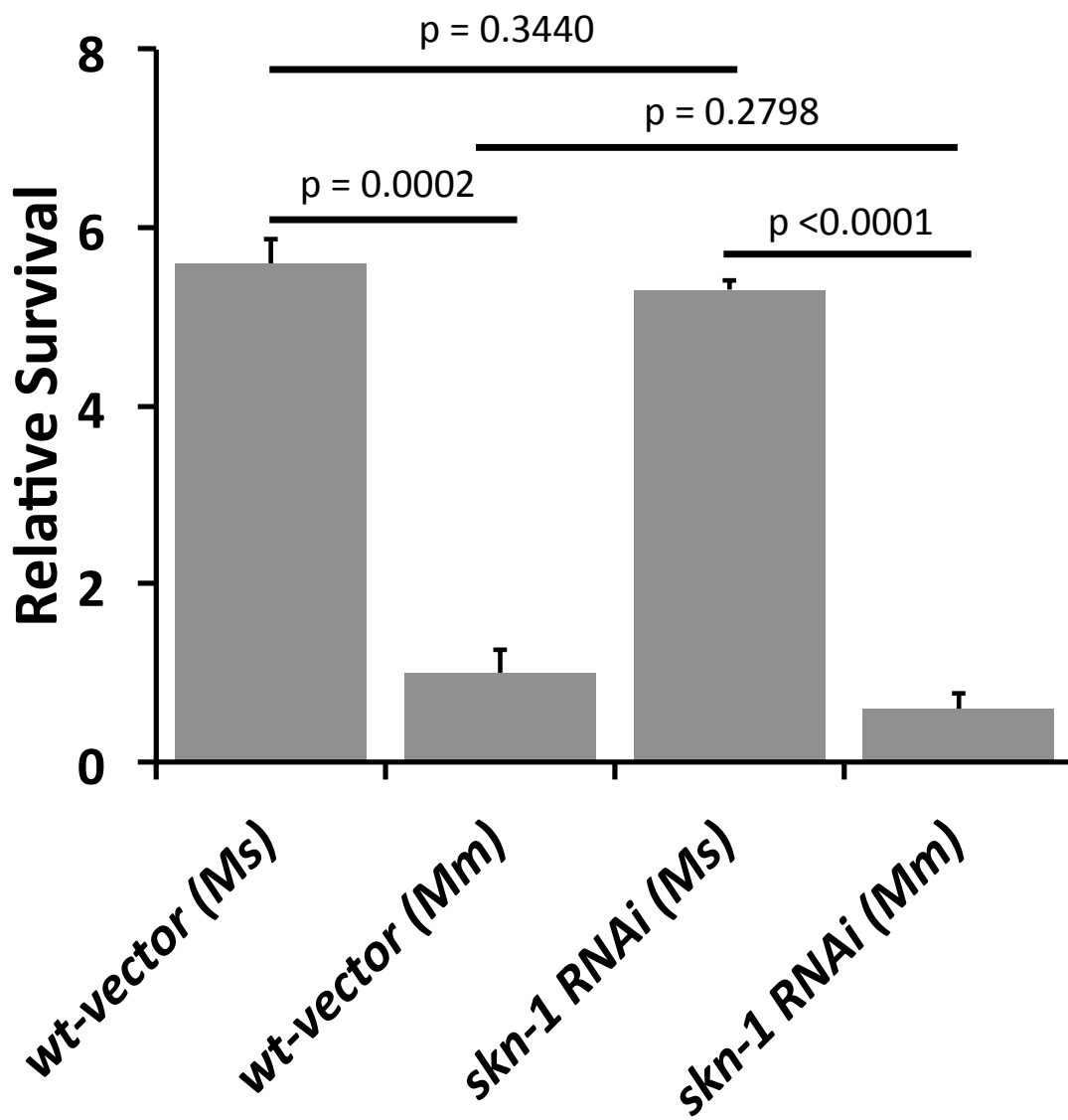

Supplement: Supplementary file 13 — Figure S13. Role of C. elegans skn‐1 in mycobacterial infection. [file MBO3-5-436-s013.pdf]

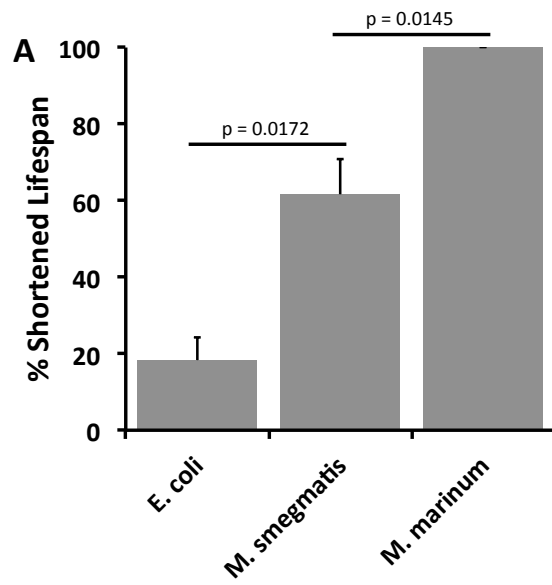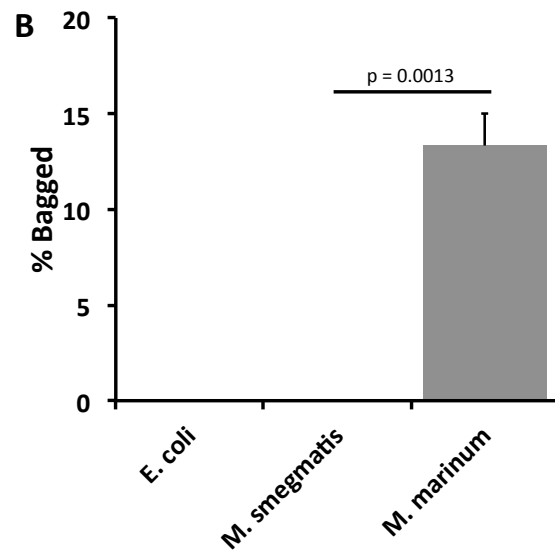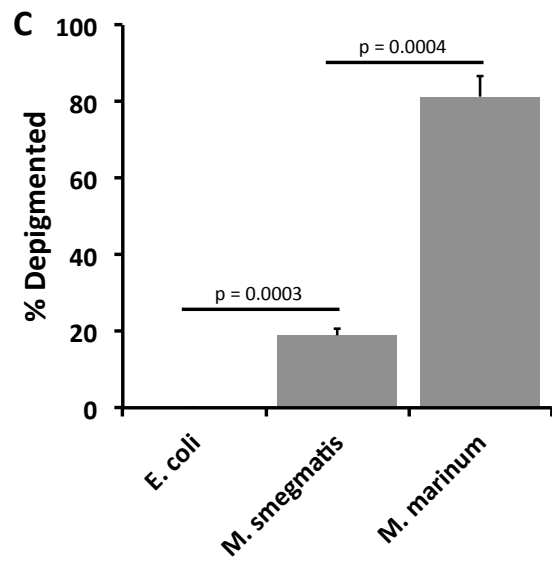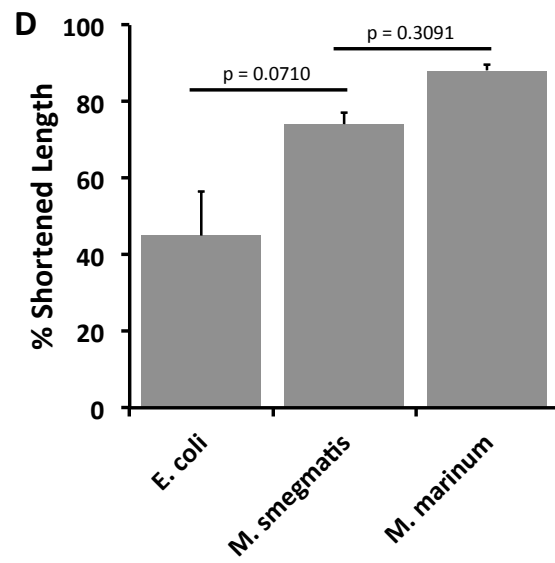

Supplement: Supplementary file 14 — Figure S14. Pathological changes in skn‐1 Mutant C. elegans infected with bacteria [file MBO3-5-436-s014.pdf]

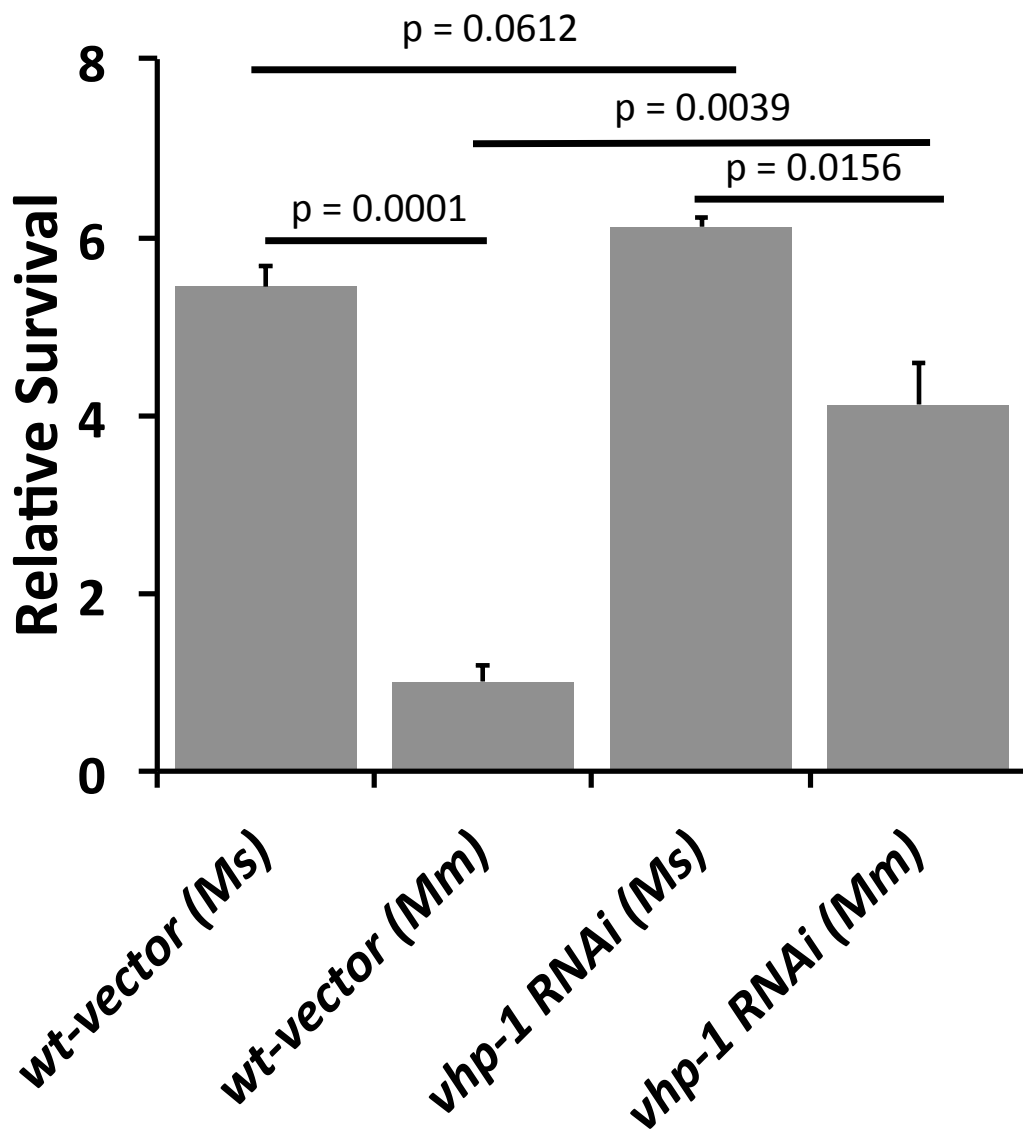

Supplement: Supplementary file 15 — Figure S15. Role of C. elegans vhp‐1 in mycobacterial infection. [file MBO3-5-436-s015.pdf]

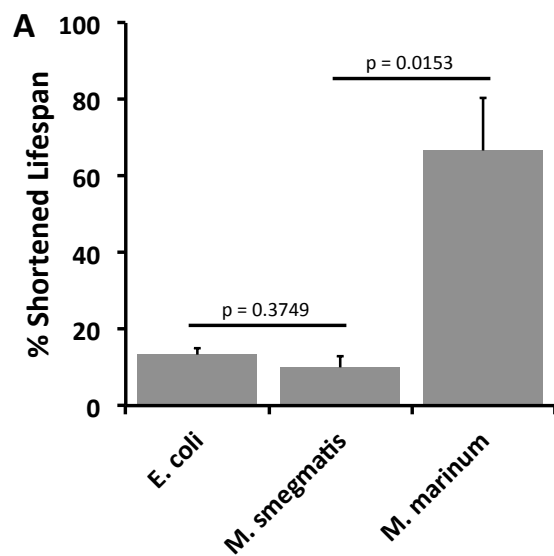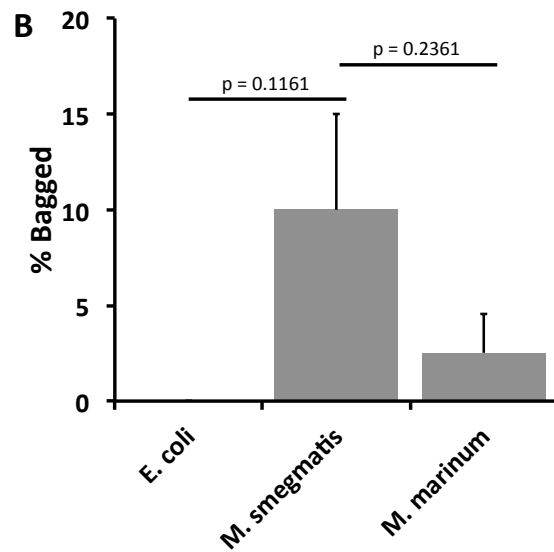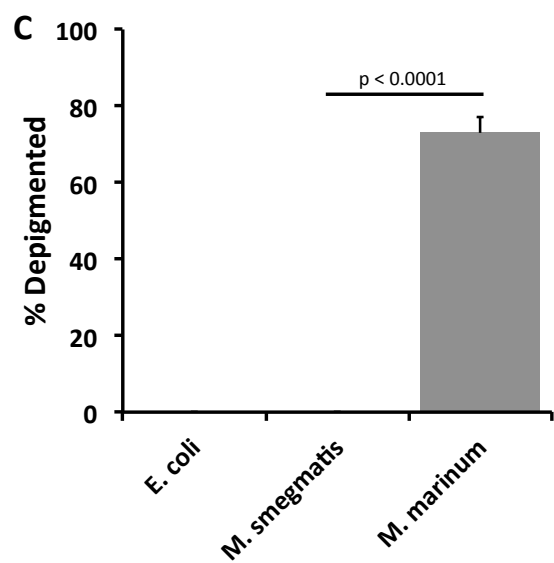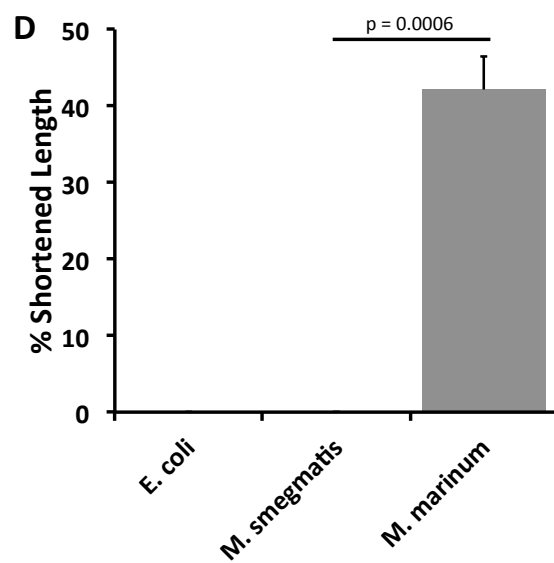

Supplement: Supplementary file 16 — Figure S16. Pathological changes in vhp‐1 Mutant C. elegans infected with bacteria. [file MBO3-5-436-s016.pdf]
