# Supplementary material for: The Caenorhabditis elegans p38 MAPK Gene plays a key role in protection from mycobacteria
Source: Microbiologyopen. 2016 Feb 25;5(3):436–52. doi: 10.1002/mbo3.341 (PMC4905996; doi:10.1002/mbo3.341)
Supplement: Supplementary file 17 — Table S1. Macrophage infection mutants (MIMs) of M. marinum. Table S2. List of C. elegans RNAi constructs and mutant strains. Table S3. Primers used for confirmation of C. elegans mutants and RNAi knock‐down. [file MBO3-5-436-s017.docx]

**SUPPLEMENTARY FIGURE LEGENDS**

Figure S1. Pathological Changes in Wild-type (N2) *C. elegans* Infected With Bacteria

(A-D) 3 trials of 20 adult N2 nematodes each (total n = 60) were infected with *E. coli* (OP50), *M. smegmatis* (mc^2^155) or *M. marinum* (ψmm1) for 24 hours, and pathological changes were characterized. P-values are shown comparing *E. coli* to *M. smegmatis* and *M. smegmatis* to *M. marinum* infection (unpaired t-test).

(A) Mean (±SEM) percentage of nematodes that died by 11 days post-infection (day 15), characterized as having a shortened lifespan.

(B) Mean (±SEM) percentage of nematodes that bagged and died by 2 days post-infection (day 6).

(C-D) For each trial, the percent of depigmentation and shortened length were characterized for the remaining nematodes after initial mortality due to bacterial infection.

(C) Mean (±SEM) percentage of nematodes that display a loss of pigmentation at 2 days post-infection (day 6), characterized by a visible reduction in cuticular pigmentation.

(D) Mean (±SEM) percentage of nematodes with a length of less than 2/3^rd^ that of a healthy adult nematode at 2 days post-infection (day 6).

Figure S2. Morphological Characteristics of *C. elegans* (TP12) Infected With *E. coli* (OP50)

Adult TP12 nematodes (expressing GFP) infected with *E. coli* (OP50) were imaged at 4 and 24 hours during infection and 6 hours post-infection (30 hours), for comparison with nematodes infected with mycobacteria for 24 hour. The head, mid-gut and lower-gut regions of five TP12 nematodes were imaged at 4, 24 and 30 hours using a confocal microscope. A 40x oil-immersion objective with a digital zoom of 2.5x (effective magnification 100x). A spectral filter for excitation wavelengths of 500-640 nm was used.

(A, D, G) Head region of the nematode displaying the oral canal and the pharyngeal pump. The shaded area beneath the pharyngeal pump is due to darker pigmentation of the nematodes.

(B, E, H) Mid-gut region of the nematode displaying fertilized ova lined up in a row in close to the vulva. The ova are in early stages of embryonic cell division. The lining of the gut is partly obstructed by the opaque dark pigmentation seen in healthy adult nematodes.

(C, F, I) Lower-gut region of the nematode displaying the tail end of the nematode and its anal opening. The lumen of the lower gut is narrow, similar to the mid-gut lumen region.

Figure S3. Survival of TP12 after Bacterial Infection

Adult TP12 nematodes (expressing GFP) were infected with *E. coli* (OP50), *M. smegmatis* (ψms23) or *M. marinum* (ψmm91) for 24 hours on day 3 as indicated by the grey shaded region in the graph (n=60). After recovering onto NGM plates seeded with OP50 nematode survival was followed. Survival of TP12 worms was similar to that of N2 worms (wild type) infected with bacteria. A log-rank method was used to compare survival of each infection group. *M. marinum* (*Mm)* and *M. smegmatis* *(Ms)* p < 0.0001; *Mm* and *E. coli* p < 0.0001; *Ms* and *E. coli* p = 0.2181.

Figure S4. Pathological Changes in TP12 *C. elegans* Infected With Bacteria

(A-D) 3 groups of 20 adult TP12 nematodes (total n = 60 per bacteria) were infected with *E. coli* (OP50), *M. smegmatis* (mc^2^155) or *M. marinum* (ψmm1) for 24 hours, and pathological changes were evaluated. P-values are shown comparing *E. coli* to *M. smegmatis* and *M. smegmatis* to *M. marinum* (unpaired t-test).

(A) Mean (±SEM) percentage of nematodes that died by 11 days post-infection (day 15).

(B) Mean (±SEM) percentage of nematodes that bagged and died by 2 days post-infection (day 6).

(C-D) For each experiment the percent depigmentation and shortened length were determined for the remaining nematodes after initial mortality due to bacterial infection.

(C) Mean (±SEM) percentage of nematodes that display loss of pigmentation at 2 days post-infection (day 6), characterized by a visible reduction in cuticular pigmentation.

(D) Mean (±SEM) percentage of nematodes with a length less than 2/3^rd^ that of a healthy adult nematode at 2 days post-infection (day 6).

Figure S5. Bacterial Load in *C. elegans* (N2) Determined by Plating for CFU

Adult N2 nematodes infected with either *M. smegmatis* (mc^2^155) or *M. marinum* (ψmm1) for 24 hours, were homogenized and plated for colony forming units (CFU) at 4, 12 and 24 hours during infection and post-infection (30 and 48 hours). 30 nematodes at each time point were homogenized and plated for CFU, except for *M. marinum* exposed nematodes at 48 hours due to their high mortality rate and the lack of nematodes requiring fewer nematodes be used. 20 worms were homogenized and plated for this point (48 hours, *M. marinum* infection). P-values are shown for comparison of CFU recovered from *M. smegmatis* and *M. marinum* infection at each time point (unpaired t-test).

Figure S6*. C. elegans* Infected With Complemented MIM of *M. marinum*

Several macrophage infection mutants (MIMs) of *M. marinum* were complemented for confirmation by introducing their orthologs *Mtb* gene in an earlier study that characterized MIMs {Mehta, 2006 #66}. Three MIMs, *mimA*, *mimG* and *mimI* and their complemented strains were compared to confirm the attenuation MIMs used in this study. The complementing genes were introduced in a plasmid vector, pJDC89. Adult N2 nematodes were infected with each respective *M. marinum* strain for 24 hours and mortality was assessed two days post-infection (day 6). P-values are shown for comparisons of the mutants and their respective complemented clones. This experiment was done twice. Complemented *M. marinum* strain of *mimA*, *mimG* and *mimI* mutants were significantly more virulent compared to their respective mutants.

Figure S7. Impact of C. elegans *pmk-1* on Infection With Mycobacteria.

Relative survival of *C. elegans* (A) MAPK (*pmk-1*) RNAi knock-down infected with *M. smegmatis* *(Ms)* and *Mm* 2 d post-infection as compared to wild-type. Relative survival of a *C. elegans* (B) MAPK (*pmk-1*) mutant infected with *Ms* and *Mm* 2 d post-infection as compared to wild-type *C. elegans*. Relative survival = number of RNAi knock-down nematodes surviving/number of wild-type surviving. Data are means and standard deviations. P values for relative survival from unpaired t-tests.

Figure S8. Pathological Changes in *pmk-1* Mutant *C. elegans* Infected With Bacteria

(A-D) 3 groups of 20 adult *pmk-1* (*km25*) mutant nematodes (total n = 60 per bacteria) were infected with *E. coli* (OP50), *M. smegmatis* (mc^2^155) or *M. marinum* (ψmm1) for 24 hours, and pathological changes were evaluated. P-values are shown comparing *E. coli* to *M. smegmatis* and *M. smegmatis* to *M. marinum* (unpaired t-test).

(A) Mean (±SEM) percentage of nematodes that died by 11 days post-infection (day 15).

(B) Mean (±SEM) percentage of nematodes that bagged and died by 2 days post-infection (day 6).

(C-D) For each experiment the percent depigmentation and shortened length were determined for the remaining nematodes after initial mortality due to bacterial infection and in the case of the C. elegans *pmk-1* mutant, no *M. marinum* infected nematodes remain, leaving only two bars on each graph.

(C) Mean (±SEM) percentage of nematodes that display loss of pigmentation at 2 days post-infection (day 6), characterized by a visible reduction in cuticular pigmentation.

(D) Mean (±SEM) percentage of nematodes with a length less than 2/3^rd^ that of a healthy adult nematode at 2 days post-infection (day 6).

**Figure S9. The *C. elegans* tol-1, dbl-1 and daf-16 Pathways Do Not Impact Mycobacterial Infection.**

Relative survival of a *C. elegans* (A) TLR (*tol-1*), TGF- β (*dbl-1*) and insulin-like receptor (*daf-16*) mutant infected with *Ms* and *Mm* 2 d post-infection as compared to wild-type *C. elegans*. Relative survival = number of RNAi knock-down nematodes surviving/number of wild-type surviving. Data are means and standard deviations. P values for relative survival from unpaired t-tests. (B) Survival curve for the *C. elegans* TLR (*tol-1*) mutant after 24 h infection. *Mm vs. Ms* p < 0.0001; *Mm vs. E. coli* p < 0.0001; *Ms vs. E. coli* p = 0.9219. Key in (B) is for (B-D). (C) Survival curve for the *C. elegans* TGF- β (*dbl-1*) mutant after 24 h infection. *Mm vs. Ms* p = 0.0001; *Mm vs. E. coli* p < 0.0001; *Ms vs. E. coli* p = 0.1939. (D) Survival curve for the *C. elegans* insulin-like receptor (*daf-16*) mutant after 24 h infection. *Mm vs. Ms* p <0.0001; *Mm vs. E. coli* p < 0.0001; *Ms vs. E. coli* p = 0.6574. Survival curve P values from log rank analysis.

Figure S10. Pathological Changes in *tol-1* Mutant *C. elegans* Infected With Bacteria

(A-D) 3 groups of 20 adult *tol-1* (*nr2033*) mutant nematodes (total n = 60 per bacteria) were infected with *E. coli* (OP50), *M. smegmatis* (mc^2^155) or *M. marinum* (ψmm1) for 24 hours, and pathological changes were evaluated. P-values are shown comparing *E. coli* to *M. smegmatis* and *M. smegmatis* to *M. marinum* (unpaired t-test).

(A) Mean (±SEM) percentage of nematodes that died by 11 days post-infection (day 15).

(B) Mean (±SEM) percentage of nematodes that bagged and died by 2 days post-infection (day 6).

(C-D) For each experiment the percent depigmentation and shortened length were characterized for the remaining nematodes after initial mortality due to bacterial infection.

(C) Mean (±SEM) percentage of nematodes that display a loss of pigmentation at 2 days post-infection (day 6), characterized by a visible reduction in cuticular pigmentation.

(D) Mean (±SEM) percentage of nematodes with a length less than 2/3^rd^ that of a healthy adult nematode at 2 days post-infection (day 6).

Figure S11. Pathological Changes in *dbl-1* Mutant *C. elegans* Infected With Bacteria

(A-D) 3 groups of 20 adult *dbl-1* (*nk3*) nematodes (total n = 60 per bacteria) were infected with *E. coli* (OP50), *M. smegmatis* (mc^2^155) or *M. marinum* (ψmm1) for 24 hours, and pathological changes were evaluated. P-values are shown comparing *E. coli* to *M. smegmatis* and *M. smegmatis* to *M. marinum* (unpaired t-test).

(A) Mean (±SEM) percentage of nematodes that died 11 days post-infection (day 15).

(B) Mean (±SEM) percentage of nematodes that bagged and died 2 days post-infection (day 6).

(C-D) For each experiment the percent depigmentation and shortened length were characterized for the remaining nematodes after initial mortality due to bacterial infection.

(C) Mean (±SEM) percentage of nematodes with loss of pigmentation 2 days post-infection (day 6), characterized by a visible reduction in cuticular pigmentation.

(D) Mean (±SEM) percentage of nematodes with a length less than 2/3^rd^ that of a healthy adult nematode 2 days post-infection (day 6).

Figure S12. Pathological Changes in *daf-16* Mutant *C. elegans* Infected With Bacteria

(A-D) 3 groups of 20 adult *daf-16* (*mgDf50*) mutant nematodes (total n = 60 per bacteria) were infected with *E. coli* (OP50), *M. smegmatis* (mc^2^155) or *M. marinum* (ψmm1) for 24 hours, and pathological changes were evaluated. P-values are shown comparing *E. coli* to *M. smegmatis* and *M. smegmatis* to *M. marinum* (unpaired t-test).

(A) Mean (±SEM) percentage of nematodes that died by 11 days post-infection (day 15).

(B) Mean (±SEM) percentage of nematodes that bagged and died by 2 days post-infection (day 6).

(C-D) For each experiment the percent depigmentation and shortened length were characterized for the remaining nematodes after initial mortality due to bacterial infection.

(C) Mean (±SEM) percentage of nematodes that display loss of pigmentation at 2 days post-infection (day 6), characterized by a visible reduction in cuticular pigmentation.

(D) Mean (±SEM) percentage of nematodes with a length less than 2/3^rd^ that of a healthy adult nematode 2 days post-infection (day 6).

**Figure S13. Role of *C. elegans* *skn-1* in Mycobacterial Infection.**

Relative survival of *C. elegans* downstream regulator of MAPK (*skn-1)* RNAi knock-down infected with *M. smegmatis* *(Ms)* and *Mm* 2 d post-infection as compared to wild-type. Relative survival = number of RNAi knock-down nematodes surviving/number of wild-type surviving. Data are means and standard deviations. P values for relative survival from unpaired t-tests.

Figure S14. Pathological Changes in *skn-1* Mutant *C. elegans* Infected With Bacteria

(A-D) 3 groups of 20 adult *skn-1* (*zu135*) mutant nematodes (total n = 60 per bacteria) were infected with *E. coli* (OP50), *M. smegmatis* (mc^2^155) or *M. marinum* (ψmm1) for 24 hours, and pathological changes were evaluated. P-values are shown comparing *E. coli* to *M. smegmatis* and *M. smegmatis* to *M. marinum* (unpaired t-test).

(A) Mean (±SEM) percentage of nematodes that died by 11 days post-infection (day 15).

(B) Mean (±SEM) percentage of nematodes that bagged and died by 2 days post-infection (day 6).

(C-D) For each experiment the percent depigmentation and shortened length were characterized for the remaining nematodes after initial mortality due to bacterial infection.

(C) Mean (±SEM) percentage of nematodes that display loss of pigmentation at 2 days post-infection (day 6), characterized by a visible reduction in cuticular pigmentation.

(D) Mean (±SEM) percentage of nematodes with a length less than 2/3^rd^ that of a healthy adult nematode 2 days post-infection (day 6).

**Figure S15. Role of *C. elegans* *vhp-1* in Mycobacterial Infection.**

Relative survival of *C. elegans* MAPK phosphatase (*vhp-1)* RNAi knock-down infected with *M. smegmatis* *(Ms)* and *Mm* 2 d post-infection as compared to wild-type. Relative survival = number of RNAi knock-down nematodes surviving/number of wild-type surviving. Data are means and standard deviations. P values for relative survival from unpaired t-tests.

Figure S16. Pathological Changes in *vhp-1* Mutant *C. elegans* Infected With Bacteria

(A-D) 3 groups of 20 adult *vhp-1* (*sa366*) mutant nematodes (total n = 60 per bacteria) were infected with *E. coli* (OP50), *M. smegmatis* (mc^2^155) or *M. marinum* (ψmm1) for 24 hours, and pathological changes were evaluated. P-values are shown comparing *E. coli* infection to *M. smegmatis* infection and *M. smegmatis* infection to *M. marinum* infection (unpaired t-test).

(A) Mean (±SEM) percentage of nematodes that died by 11 days post-infection (day 15).

(B) Mean (±SEM) percentage of nematodes that bagged and died by 2 days post-infection (day 6).

(C-D) For each experiment the percent depigmentation and shortened length were characterized for the remaining nematodes after initial mortality due to bacterial infection.

(C) Mean (±SEM) percentage of nematodes that display loss of pigmentation at 2 days post-infection (day 6), characterized by a visible reduction in cuticular pigmentation.

(D) Mean (±SEM) percentage of nematodes with a length less than 2/3^rd^ that of a healthy adult nematode 2 days post-infection (day 6).

# SUPPLEMENTARY Tables

Table S1. Macrophage infection mutants (MIMs) of *M. marinum.*

| Gene^1^ | Putative activity^2^ | *Mtb^3^ homolog*^4^ | *Mm* Genome^5^ |
| --- | --- | --- | --- |
| *fadD29* | fatty acid-CoA ligase | Rv2950c | *fadD29* |
| *fadD30* | fatty acid-CoA ligase | Rv0404 | *fadD30* |
| *mimA* | membrane protein | Rv0246 | MMAR_0508 |
| *mimB* | integrase/recombinase | None | MMAR_3589 |
| *mimC* | o-phosphotransferase | Rv2636 | MMAR_2065 |
| *mimD* | hypothetical protein | None | *mimD* |
| *MimE* | copper amine oxidase | None | *maoX* |
| *mimF* | phage protein | None | MMAR_3906 |
| *mimG* | Amidophosphoribosyltransferase | Rv3242c | MMAR_1303 |
| *mimH* | secretion (extRD1) | Rv3881c | MMAR_0202 |
| *mimI* | hypothetical protein | Rv1502 | MMAR_2318 |
| *mimJ* | hypothetical protein | None | *mimJ* |
| *mimK* | hypothetical protein | None | MMAR_4589 |
| *Nrp* | glycopeptidolipid synthesis | Rv0101 | MMAR_3779 |
| *pks12* | polyketide synthesis | Rv2048c | MMAR_0101 |
| *ppe24* | PPE24 | Rv1753c | MMAR_1028 |
| *ppe53* | PPE53 | Rv3159c | MMAR_4554 |
| *sdhD* | succinate dehydrogenase | Rv3317 | *sdhD* |

^1^Mutants as constructed by Mehta et al.

^2^Putative activities based on similarity from the National Center for Biotechnology Information.

^3^*Mycobacterium tuberculosis*

^4^Homolog based on current annotation.

^5^*Mycobacterium marinum* genome annotation designation NCBI (Accession: NC_010612.1).

Table S2. List of *C. elegans* RNAi constructs and mutant strains.

| Gene | RNAi Construct^1^ | Mutant Strain^2^ | Mutant Genotype |
| --- | --- | --- | --- |
| *daf-16* | R13H8.1 | GR1307 | *daf-16*(*mgDf50*) |
| *dbl-1* | T25F10.2 | NU3 | *dbl-1*(*nk3*) |
| *pmk-1* | B0218.3 | KU25 | *pmk-1*(*km25*) |
| *skn-1* | T19E7.2 | EU31 | *skn-1*(*zu135*) |
| *tol-1* | C07F11.1 | IG10 | *tol-1*(*nr2033*) |
| *vhp-1* | F08B1.1 | JT366 | *vhp-1*(*sa366*) |

^1^RNAi constructs were cloned into pL4440-DEST that expresses resistance to ampicillin (100 µg/ml). The *E. coli* host strain is HT115 (DE3) (obtained from the laboratory of Dr. Andrew Fire).

^2^Mutant strains were obtained from *Caenorhabditis* Genetics Center (CGC), University of Minnesota.

Table S3. Primers used for confirmation of *C. elegans* mutants and RNAi knock-down.

| Gene | Forward primers^1^ Reverse primers^1^ | | Product length |
| --- | --- | --- | --- |
| *pmk-1* | AACTGGAACACCAGATGAAG | TTGAAATCACGGCGAGTC | 99bp |
| *daf-16* | CGGTTCCAGCAATTCCA | GCTTCGACTCCTGCTTAAT | 102bp |
| *dbl-1* | ACTGAGCTCATTGCCCTA | TTTGTGCACTCCGTTTCC | 101bp |
| *tol-1* | CACAGCTTCATCGGAAGAC | AGTGCTGGAAGAGTATGGA | 98bp |
| *vhp-1* | TGTTGTCGAGAACCCATTT | GCAGATGCTGGAGTTGAT | 96bp |
| *skn-1* | CGTCAACAGCAGACTCAAA | CGAGTGTCTCTGTGAGTGA | 96bp |
| *cdc-42* | GCTCGAGAAACTGGCAAA | CGCTGAGCATTCAACGTA | 103bp |
| *pmp-3* | TCGCTAACTGAATGGAGAAT | TGATGAACACGGGAACAC | 103bp |

^1^Forward and Reverse primers were selected using Oligo Analyzer Tools available on the Integrated DNA Technologies website, to have a melt temperature of 60ºC.
